# Soil management strategies drive divergent impacts on pathogens and environmental resistomes

**DOI:** 10.1038/s41598-025-27157-9

**Published:** 2025-12-05

**Authors:** Colette A. Nickodem, Patricia Q. Tran, Eric Neeno-Eckwall, Afton G. Congdon, Gregg R. Sanford, Erin M. Silva, Jessica L. Hite

**Affiliations:** 1 Department of Pathobiological Sciences, School of Veterinary Medicine, University of Wisconsin-Madison, Madison, WI USA; 2 Department of Bacteriology, University of Wisconsin-Madison, Madison, WI USA; 3 College of Agriculture & Life Sciences, University of Wisconsin-Madison, Madison, WI USA; 4 Department of Soil & Environmental Sciences, University of Wisconsin-Madison, Madison, WI USA; 5 Department of Plant Pathology, University of Wisconsin-Madison, Madison, WI USA

**Keywords:** Organic, Non-organic, Synthetic fertilizers, Poultry manure, Antimicrobial resistance, Environmental sciences, Microbiology

## Abstract

Antimicrobial resistance (AMR) is a growing global health threat, and the genes that confer drug resistance are increasingly recognized as widespread environmental contaminants. Livestock manure, widely used as a non-synthetic fertilizer, is a potential source of AMR contamination in the environment. Manure fertilizers are well-documented reservoirs of AMR genes (ARGs) and drug-resistant pathogens. However, the role of soil management practices in shaping the persistence and spread of these genes after manure application remains poorly understood. We conducted a large-scale field experiment to evaluate how soil management practices influence the resistome (the genomic content involved in resistance to antimicrobial agents) and the overall microbiome of agricultural soils. Specifically, we ask: Does the use of composted poultry manure in organic soil management practices increase the *risk* of transmitting ARGs and drug-resistant pathogens? We integrated metagenomic sequencing with risk score analyses to assess the abundance, diversity, and mobility of resistance genes. Contrary to expectations, our results indicate that non-organic practices, despite not applying poultry manure, posed greater risks for transmitting AMR genes and human pathogens — due to significantly higher co-occurrence of ARGs with mobile genetic elements (MGEs), which facilitate horizontal gene transfer. In contrast, organic practices, that applied composted poultry manure, increased overall ARG and metal resistance gene (MRG) abundance, but the genes were less diverse and less mobile. These findings show that focusing solely on ARG and MRG abundance can misrepresent AMR risks and underscore the importance of evaluating gene mobility and management context when assessing AMR hazards. Our study highlights how soil management can be strategically leveraged to mitigate AMR transmission, offering actionable insights for sustainable agriculture, environmental stewardship, and public health protection.

**Supplementary Information:**

The online version contains supplementary material available at 10.1038/s41598-025-27157-9.

## Introduction

The overuse of antimicrobials in human and animal systems is a major driver of antimicrobial resistance (AMR) and a growing global health threat^1–3^. Livestock production accounts for approximately 73% of all antibiotic use worldwide^4^. Although few of the antimicrobials used in livestock are identical to those used in human medicine, many are structurally similar, raising concern about the development of broad-spectrum drug resistance^5,6^. Stewardship efforts have helped reduce antimicrobial use in agriculture. These strategies, however, have been unable to prevent the rise in antimicrobial resistance or the emergence and spread of antimicrobial resistance genes (ARGs) within and across agricultural systems^2,7^. Indeed, AMR is increasingly recognized not only as a clinical issue but also as a critical environmental contaminant with unknown impacts on natural ecosystems and public health^8,9^. While ARGs within microbial communities are not inherently harmful to human health, these genes can transfer between non-pathogenic and pathogenic bacteria, increasing the risk of drug-resistant infections^10^.

One possible source of environmental AMR contamination is livestock manure, which is commonly applied to agricultural fields as a fertilizer. Manure is a well-documented hotspot for ARGs, zoonotic pathogens, and mobile genetic elements (MGEs), driven by its high microbial density, nutrient richness, and the presence of selective agents such as antimicrobials and heavy metals^11–14^. Yet, the persistence and risks of these pathogens, ARGs, and MGEs in agricultural fields are shaped by other management practices such as crop variety, crop rotations, tillage, herbicide use, and other factors that together shape the soil microbiome^15–17^. These complex interactions raise the intriguing possibility that soil management practices could be leveraged to help reduce the environmental resistome (the genomic content involved in resistance to antimicrobial agents) and the spread of drug-resistant pathogens. However, while considerable research has focused on pre-application manure treatment strategies to reduce risks associated with manure fertilizers, few studies have examined how soil management strategies shape the downstream impacts of manure fertilizers^18–21^.

Here, we focus on links between soil management and poultry manure, which is widely used as a non-synthetic fertilizer. While poultry manure provides agronomic benefits such as improved soil fertility and organic matter input, it could also introduce ARGs, MGEs, heavy metals, and zoonotic pathogens that persist and proliferate in the environment^12,22–27^. Numerous studies have linked antimicrobial resistance to poultry production systems and examined how pre-application processing and composting shift patterns of AMR genes^28,29^. Yet, the extent to which poultry manure application contributes to the spread of pathogens and antimicrobial resistance genes to soil and crops in real agricultural systems remains underexplored. Even less is known about relationships between ARGs, metal resistance genes (MRGs), and MGEs that have the potential to disseminate these contaminants via horizontal gene transfer, facilitating their movement to new microbial hosts. Without these insights, our understanding remains limited to identifying localized resistance hot spots, rather than advancing toward proactive, evidence-based strategies that pinpoint critical control points for mitigating onward transmission.

Our systems-based approach directly confronts these limitations by examining the role of poultry manure within the broader management context in which it is applied. Specifically, we examine these dynamics within realistic field conditions that reflect industry-standard organic cropping systems and compare these practices to non-organic cropping systems, which rely on synthetic fertilizers. We do this first by reviewing the current literature and understanding of how poultry manure impacts the environmental microbiome and resistome. Then, we integrate high-resolution genomic tools to assess how poultry manure influences the abundance, diversity, and transmission potential (risk) of drug-resistant pathogens in soil. We apply risk score analyses using recently developed bioinformatic tools. For example, the AMR + + pipeline, which efficiently identifies microbial taxonomy and antimicrobial resistance gene profiles but does not identify mobile genetic elements, misses a critical component for AMR transmission^30,31^. Our approach goes further, by integrating risk score analyses to quantify the likelihood of drug-resistant pathogens spreading through host-environment and host-host transmission pathways. Specifically, we use MetaCompare 2.0 to identify MGEs in proximity to ARGs within bacterial genomes to quantify the risk of ARG transfer^32^. Applying these bioinformatic resources to environmental sequencing data will provide a more comprehensive overview of AMR pathogen transmission risk across complex environments.

### Why focus on poultry manure? A brief review

As an alternative to synthetic fertilizer, the use of poultry manure provides an excellent opportunity to enhance soil health in cropping systems and promote environmental sustainability. Poultry manure can improve soil fertility by increasing organic matter, enhancing microbial activity, improving nutrient availability, and contributing to carbon sequestration^33–35^. Additionally, the use of poultry manure as fertilizer offers a practical solution to this copious by-product. Over 14 million tons of chicken litter are produced annually in the U.S^34^. A single broiler house accommodating 20,000 birds can produce approximately 150 tons of litter per year, and a flock of 14,000 breeder hens can yield a similar amount^36^. Thus, manure fertilizers offer an opportunity to transform agricultural waste into a valuable resource for soil productivity.

However, poultry manure can also serve as a reservoir for zoonotic pathogens and antimicrobial resistance genes^37^. Poultry can shed significant quantities of pathogens through feces to litter including, *Campylobacter spp.*, *Salmonella enterica*, *and Escherichia coli*^29,38,39^. These (and other) pathogens can survive and proliferate outside their hosts in environmental reservoirs, posing zoonotic risks through indirect (environment-host) transmission^40–42^. Even when pathogens persist but do not proliferate in the environment, environmental transmission can still facilitate pathogen invasion and sustain long-term persistence within a host population^43–45^. The persistence of these pathogens in the environment not only increases the risk of zoonotic spillover but may also facilitate the spread of antimicrobial resistance.

A limited number of field and microcosm studies have shown that the risk of transmitting zoonotic pathogens and ARGs from poultry litter and raw (pig) manure treated soils to crops and fresh produce poses a public health threat^26,46,47^. Fresh poultry manure most commonly harbors antibiotic resistance genes conferring resistance to aminoglycosides (*ant(3’)-Ia*,* aph(3’)-Ia*,* aph(3’)-IIa*,* aph(6’)-Ia*), macrolides (e.g., *ermB*, *ermF*) and tetracyclines (*tet*A, *tet*G, *tet*M, *tet*Q, *tet*S, *tet*W, *tet*X), while sulfonamide (*sul1*,* sul2*) and chloramphenicol (*fexA*,* fexB*,* cfr*,* cmlA*,* floR*) resistance genes are also prevalent^12,22^. To reduce food safety risks, the USDA’s National Organic Program standards require that the application of raw manure must be applied 120-days prior to cultivating crops that have contact with the soil and 90-days prior to cultivating crops that don’t have contact with the soil^48^. Although raw poultry manure can be used in the U.S. according to these standards, most poultry manure is composted to reduce the spread of AMR pathogens.

To reduce the potential risks associated with poultry manure fertilizers, efforts have focused on pre-application methods including composting and pelletization. Poultry manure fertilizers that are made from litter are primarily composed of wood shavings used for bedding and excreta absorption. These materials are dried and pelletized to optimize nutrient delivery in fertilizer applications. Multiple rounds of composting can significantly decrease the diversity and abundance of bacteria and antibiotic residues^12^. Yet, the effectiveness of mitigating antimicrobial resistance varies significantly^49^. For example, composting can increase certain ARGs like *sul*1 and *tet*M, while decreasing others, such as *erm*B and *aac*(6’)-Ib-cr^12,22^. Further, more complex composting methods including the addition of biochar, nanoscale zero-valent iron supplementations, heat treatments, or variations (aerobic, anaerobic, and vermi) of composting have different impacts on pathogens and ARGs^49–53^. Thus, manure treatment methods do not uniformly reduce ARG levels, thereby influencing the persistence of these genes in soils amended with composted poultry manure.

How poultry manure amendments influence the prevalence, abundance, diversity, and temporal dynamics of ARGs within treated soils varies considerably across studies. While some studies indicate that poultry litter contributes minimally to microbial contamination, long-term soil applications have been linked to significant increases in ARG abundance, particularly of *sul1*,* ermB*, and *tetM*^12,14,22–25,37^. Additionally, the resistome profiles of soils amended with poultry manure highlight substantial variation linked to bird age, management practices, antimicrobial usage, and heavy metals^12,22,26^. Metals such as Zinc (Zn), Copper (Cu), Manganese (Mn), and Iron (Fe) are frequently used as food additives in the poultry industry and are often identified in poultry manure and amended soils^12,22,27^. Composting is ineffective at reducing heavy metals that contribute to antibiotic co-resistance and are strongly correlated with ARGs. For example, Cu shows a particularly strong association with macrolide, quinolone, tetracycline, beta-lactam, and aminoglycoside resistance that includes genes such as *erm*C, *qnr*S, *tet*M, *bla*CTX-M, and *aac*(6’)-Ib-cr^12,24^. Together, these studies suggest that agrochemicals, including antimicrobials and heavy metals, can act synergistically to co-select for AMR genes.

In addition to co-selection, these agrochemicals can also contribute to the emergence of AMR by altering the soil microbiome, reducing competitive suppression from other microbes and creating novel ecological niches that favor the proliferation of drug-resistant pathogens. For instance, poultry manure amendments have been shown to reduce overall microbial diversity, fostering a narrower set of ARGs and promoting the proliferation of resistant pathogens^27^. Additionally, elevated concentrations of heavy metals such as Cu, Mn, and Zn are negatively associated with microbial alpha diversity, potentially leading to a dominance of pathogenic strains^12^.

Moreover, studies indicate that poultry manure amendments can shift microbial community composition, favoring Acidobacteria and Firmicutes while reducing Bacteroidetes, a shift that may have consequences for nutrient cycling and microbial interactions within the soil ecosystem^12^. Changes in beta diversity — or the differences in microbial community composition between environments — also suggest that manure application introduces microbial taxa that are more resistant to environmental stressors, further contributing to AMR persistence^54,55^. Therefore, understanding how management practices shape microbial interactions and influence the ecological dynamics of drug-resistant bacteria in complex communities is critical for predicting and mitigating the emergence and spread of antimicrobial resistance^56^. This more integrated approach is essential to moving beyond identifying resistance hot spots and toward a mechanistic understanding of how agricultural management shapes the ecological and evolutionary dynamics of AMR across agroecosystems.

## Methods

### Field study

This field study was conducted as part of the Wisconsin Integrated Cropping Systems Trial (WICST) at the Arlington Agricultural Research Station in Wisconsin, USA (43°20′N, 89°21′W)^57^. Established in 1989, WICST is a large-scale (24 hectares), long-term field experiment designed to reflect a range of conventional and alternative farming practices representative of those used in North Central United States and Northern Europe^58,59^. The trial incorporates diverse land management strategies, crop rotations, cover crop varieties, and includes one of the longest running organic research trials in the U.S.^57^. Unlike traditional agronomic experiments that isolate individual management components, WICST employs a systems-based approach to compare integrated farming systems. This design acknowledges the complexity of agricultural decision-making, recognizing that farmers manage interconnected sets of practices rather than discrete inputs in isolation^58,59^.

The field plots, each measuring 170 × 20 m (0.34 hectares), were designed to accommodate full-scale farm equipment, ensuring that the study accurately reflects on-farm conditions^58–63^. The trial follows a randomized complete block design, incorporating both spatial and temporal randomization. Each crop phase within a system is replicated across four blocks, and a staggered start ensures that every phase is represented annually. This experimental design enhances the capacity to capture annual weather variability across cropping phases, while also improving statistical robustness for long-term trend analyses. However, the scale and complexity of the trial design comes with trade-offs. Notably, the absence of paired controls for each individual treatment limits the ability to isolate the effects of specific management practices. On the other hand, implementing such controls would be impractical, as no single crop or treatment can serve as a universal comparator across diverse systems. For these reasons, the integrated, systems-based approach we employed here is standard for investigating complex agricultural systems^64,65^.

In this study, we focused on two cropping systems from WICST: a non-organic system (NO), which received synthetic fertilizer, and an organic system (O), which received composted poultry manure (1.5 ton/ac) once in April. Although we focus on fertilizer types in this study, these cropping systems differ in additional management practices. The non-organic system represents a conventional continuous corn cycle. Fertility in this system is derived from applied synthetic fertilizers; starter fertilizer and anhydrous ammonia, with nitrogen application rates adjusted according to preplant nitrate testing (PPNT) and in alignment with standard agronomic recommendations^64,65^. Weed control is achieved through a combination of tillage and herbicides. The organic system is also used to cultivate corn but uses a grain rotation (corn-soybean-wheat)  and is managed in compliance with the USDA National Organic Program regulations^63^.

For the organic system, fertility is derived via composted poultry manure, biologically derived N, and the incorporation of oats and berseem clover, a leguminous cover crop used as a green manure to enhance soil fertility as part of the rotation. The organic system receives minimal tillage and no herbicides. For consistency (and due to logistical constraints), we sampled only the plots that were in their corn rotation phase. Importantly, these additional soil management practices represent potential confounders in the investigation of fertilizer impact that are not explicilty accounted for in the randomized complete block design of this field study. Given that we focus here on a systems-based approach to understanding these various practices as a sum of their parts, these variables are inherently accounted for by the treatment groupings (to the best of our ability) in our statistical analyses.

### Sample collection

Sampling events followed the on-farm schedule which included sampling 1-day post-harvest and 12-days post-harvest of corn for both the organic and non-organic plots. Five samples were collected from each plot in a standard “W” formation. Sample locations were measured and recorded to ensure soil samples were taken within 1 sq ft of the original sample point at each sample event. A single soil sample consisted of a 25 g core, taken between 0 and 15 cm of the surface using a soil probe. The soil probe was cleaned with a bleach solution and 70% ethanol between each sample. The site is characterized by Plano silt loam soil (*Fine-Silty*, *Mixed*, *Superactive*, *Mesic Typic Argiudolls*) and experiences a 30-year mean annual temperature of 6.9 °C, with an average annual precipitation of 869 mm, of which 637 mm falls during the April–October cropping season^66^. During our sample collection period the average air temperature was 6.1 °C, with a maximum temperature of 25.9 °C and minimum temperature of -7.3 °C.

All samples were placed on ice and transported to the lab at the University of Wisconsin (UW) - Madison where they were stored in the refrigerator overnight. A set of each individual sample was saved: (1) a raw sample was saved in 5 ml polypropylene tubes for downstream molecular work, and (2) a portion of each sample was mixed with an equivalent volume of 50% glycerol to maintain cellular integrity for future microbiological work. All samples were stored in the freezer at -80 °C within 24 h.

### DNA extraction and sequencing

Total genomic DNA was extracted from soil samples using Qiagen PowerSoil Pro Kits (Qiagen; Hilden, Germany). DNA samples were pooled per field plot, resulting in four samples per cropping system treatment at each collection time point. DNA was then diluted to 10 ng/µl and submitted to the UW-Madison Biotechnology Center (UWBC) Sequencing Core for library preparation. DNA libraries were shotgun sequenced on an Illumina NovaSeq using 2 × 150 bp chemistry for paired-end sequencing. To achieve approximately 70–80 million reads per sample, seven lanes of 10B flow cells were used.

### Bioinformatic analyses

Raw sequences were concatenated by sample for all flow cell lanes (1 through 7). These FASTQ files were then transferred to the UW-Madison Center for High Throughput Computing (CHTC) system, which maintains cores for expedited computing^66^. A DAGMan (Directed Acyclic Graph Manager) workflow was created to run sequences through all bioinformatic analyses sequentially. First, the AMR + + v3.0 pipeline was used to identify microbial taxonomy and resistance genes^67^. This pipeline trimmed adapters and low-quality reads with Trimmomatic prior to the removal of host genome sequences^68^. We curated a host genome file containing genomes for human (*Homo sapiens*) (GCF_000001405.40), chicken (*Gallus gallus*) (GCF_016699485.2), maize (*Zea mays*) (GCF_902167145.1), soybean (*Glycine max*) (GCF_000004515.6), winter wheat (*Triticum aestivum*) (GCF_018294505.1), berseem clover (*Trifolium alexandrinum*) (GCA_043005315.1), and oat (*Avena sativa*) (GCA_916181665.1) for alignment and removal of host-associated genome sequences.

First, microbiome counts were obtained via k-mer matching to the Kraken2 database for taxonomic classification^68^. Resistome counts for antimicrobials, antibiotics, biocides, and metals were obtained through sequence alignment to the MEGARes database^69,70^. Optional flags were used to de-duplicate MEGARes alignment counts and verify ARG single nucleotide polymorphisms (SNPs). Secondly, the workflow assembled contigs using the SPAdes metagenomic option and annotated genes using Prodigal^71^. Lastly, MetaCompare 2.0 was used to identify mobile genetic elements and assign resistome risk scores. The MGEs were identified using the mobileOG-db and the best hits were selected for classification into major functional categories^71^. MetaCompare 2.0 identified contigs with annotated ARGs, ARGs + MGEs, ARGs + Pathogens (aligned), and ARGs + MGEs + Pathogens (aligned). These values were divided by the total number of contigs to serve as the proportions for 3D hazard spaces. The resistome risk scores were calculated as follows: Risk Score = (1-d_s_/d_w_) x 10^4^, where d_s_ = the Euclidean distance of the sample to the maximal point and d_w_ = the Euclidean distance of a sample containing no ARGs. MetaCompare 2.0 produced risk scores for ARG transmission of ecological pathogens (generalized environmental ARG mobilization across a range of non-ESKAPE pathogens) and human pathogens (limited to Class 1 ARGs and ESKAPE pathogens that are pertinent to human infections)^72^. These ESKAPE pathogens, designated by the World Health Organization (WHO), include *Enterococcus faecium*, *Staphylococcus aureus*, *Klebsiella pneumoniae*, *Acinetobacter baumannii*, *Pseudomonas aeruginosa*, and *Enterobacter* spp.

### Statistical analyses

Taxonomic and resistance gene count matrices from AMR + + were imported into R version 4.4.2 for further processing and statistical analysis^73^. The count matrices were converted into phyloseq objects to explore evenness, richness, and diversity using the phyloseq package^74^. Specifically, Shannon’s Index and observed richness were used to analyze alpha diversity, and the Wilcoxon rank-sum test was used to determine significance within different cropping system groups. Taxonomic data from the Kraken2 analytical matrix was first assessed using unclassified microbiome data, and then later aggregated to phylum, class, genus, and species levels. We then converted the phyloseq object to metagenomeSeq to conduct cumulative sum scaling (CSS) normalization at phylum, class, and species levels to plot normalized and relative abundances^75^. CSS normalized abundance data were also used for ordination with Bray-Curtis distance measures for non-metric multidimensional scaling (NMDS) plots^75^.

To evaluate the goodness-of-fit of the ordination we used a stress plot (Shepard plot). To determine the significant differences between cropping system groups and collection time points using the Bray-Curtis distance measures, we used ADONIS2 (PERMANOVA) to conduct a post hoc analysis of pairwise group comparisons. The model for these included cropping system group and time interaction as fixed effects with cropping system block as a random effect. We explored the impact of soil fertilization on the soil microbiomes based on categorical metadata, including indices for fertilizer, biocide integration, crop rotation complexity, crop rotation species richness, soil stability, and a soil tillage intensity rating. These variables were inherently included in the cropping system group and shared the same statistical impact.

Resistance data were processed similarly. The de-duplicated and SNP confirmed count matrix was first subset by resistance type: drug resistance, metal resistance, biocide resistance, and multi-compound resistance. Then data were aggregated to the class, mechanism, and gene group levels of resistance classification for improved comprehension.

## Results

### Sequencing metrics

To improve the accuracy and reliability of variant calling, we conducted deep sequencing for this project, resulting in 1.8 billion reads, with an average of 90 million (range: 76 M–138 M) reads per soil sample. 1.87% of reads were determined to be low quality during the adapter trimming process, and 28.1% of reads were removed during host alignment, leaving 70.03% of the original reads available for analyses.

Within soil samples, there were 96,881,598 reads classified at the phylum level and 58,951,405 reads classified at the species level. Out of the phylum-level counts, 60.8% were also classified at the species-level for soil samples, suggesting there is sufficient data (above the 50% threshold) for species-level interpretations of these samples.

### Microbiome & resistome – richness and diversity

**Fig. 1:**
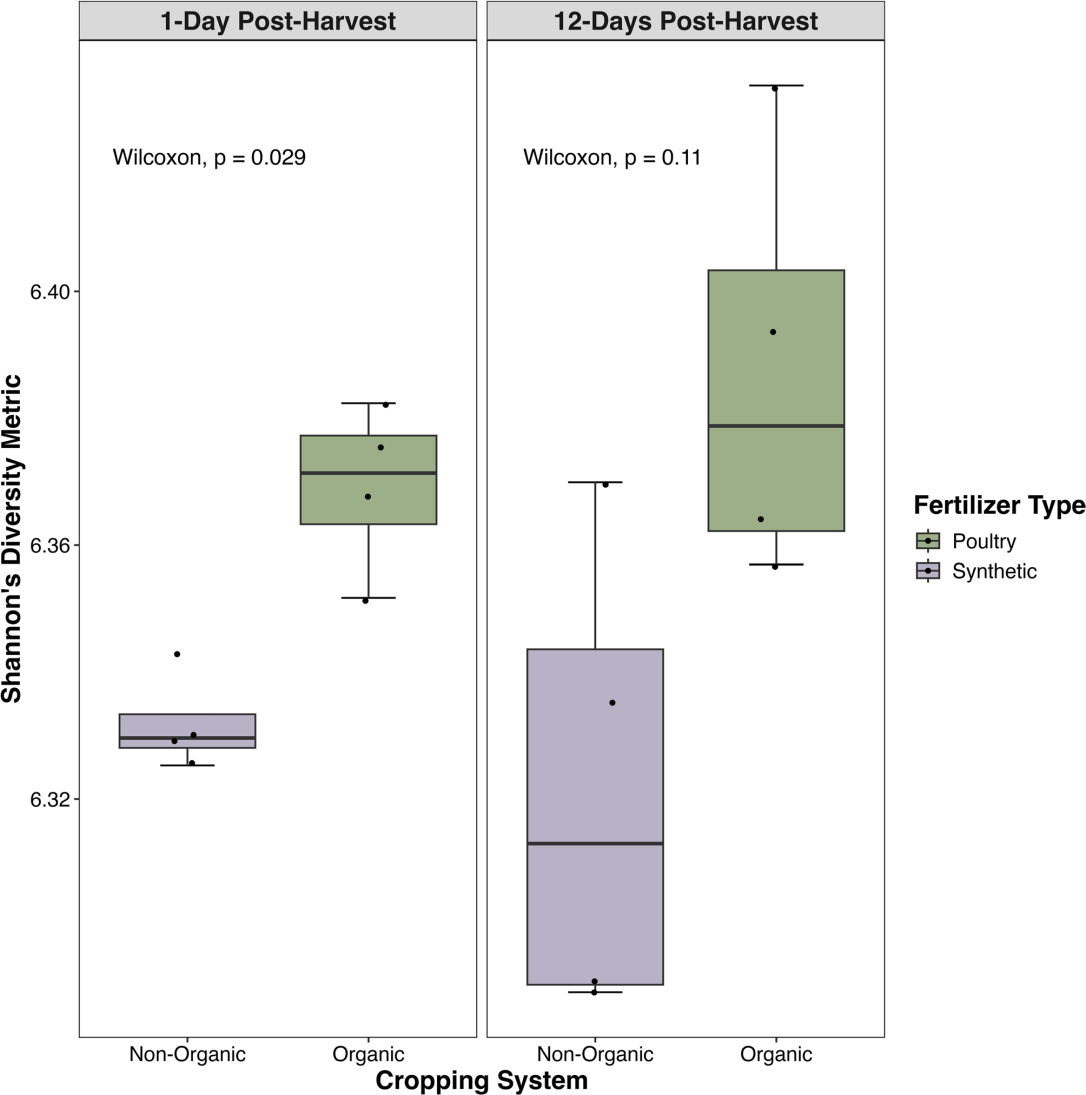
Microbiome alpha diversity by cropping system. Shannon’s diversity index indicated a significant increase within organic soils, which received composted poultry manure (green), compared to non-organic soils, which received synthetic fertilizer (purple), 1-day post-harvest, (Wilcoxon test: *P* = 0.03) but not 12-days post-harvest (Wilcoxon test: *P* = 0.11).

**Fig. 2:**
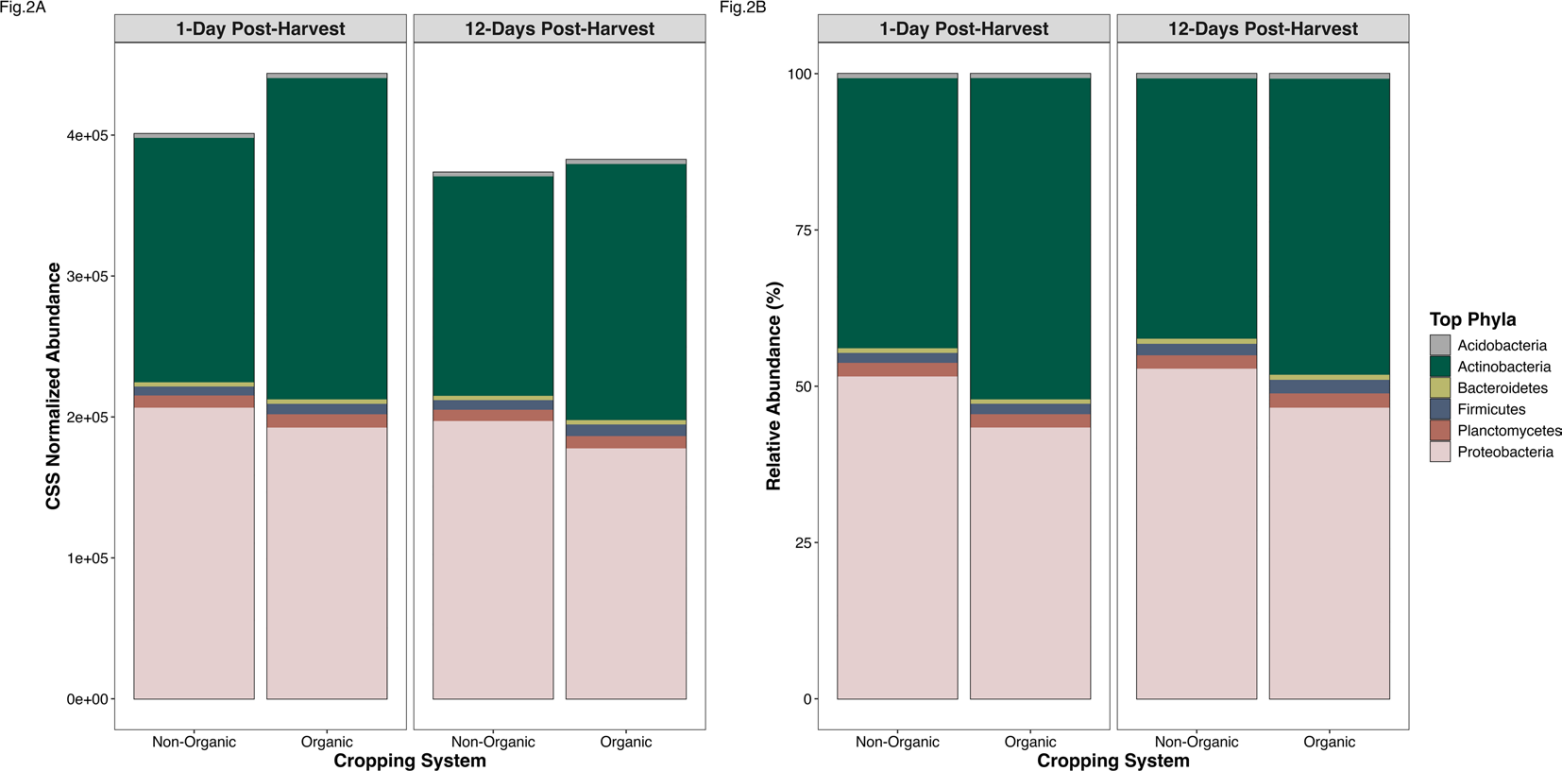
Top abundant phyla of the microbiome. Phyla that constitute the top 0.5% most abundant phyla of non-organic and organic cropping system soils by 1-day and 12-days post-harvest. **(A)** Cumulative sum scaled (CSS) normalized abundance of top phyla. **(B)** Relative abundance of top phyla.

**Fig. 3:**
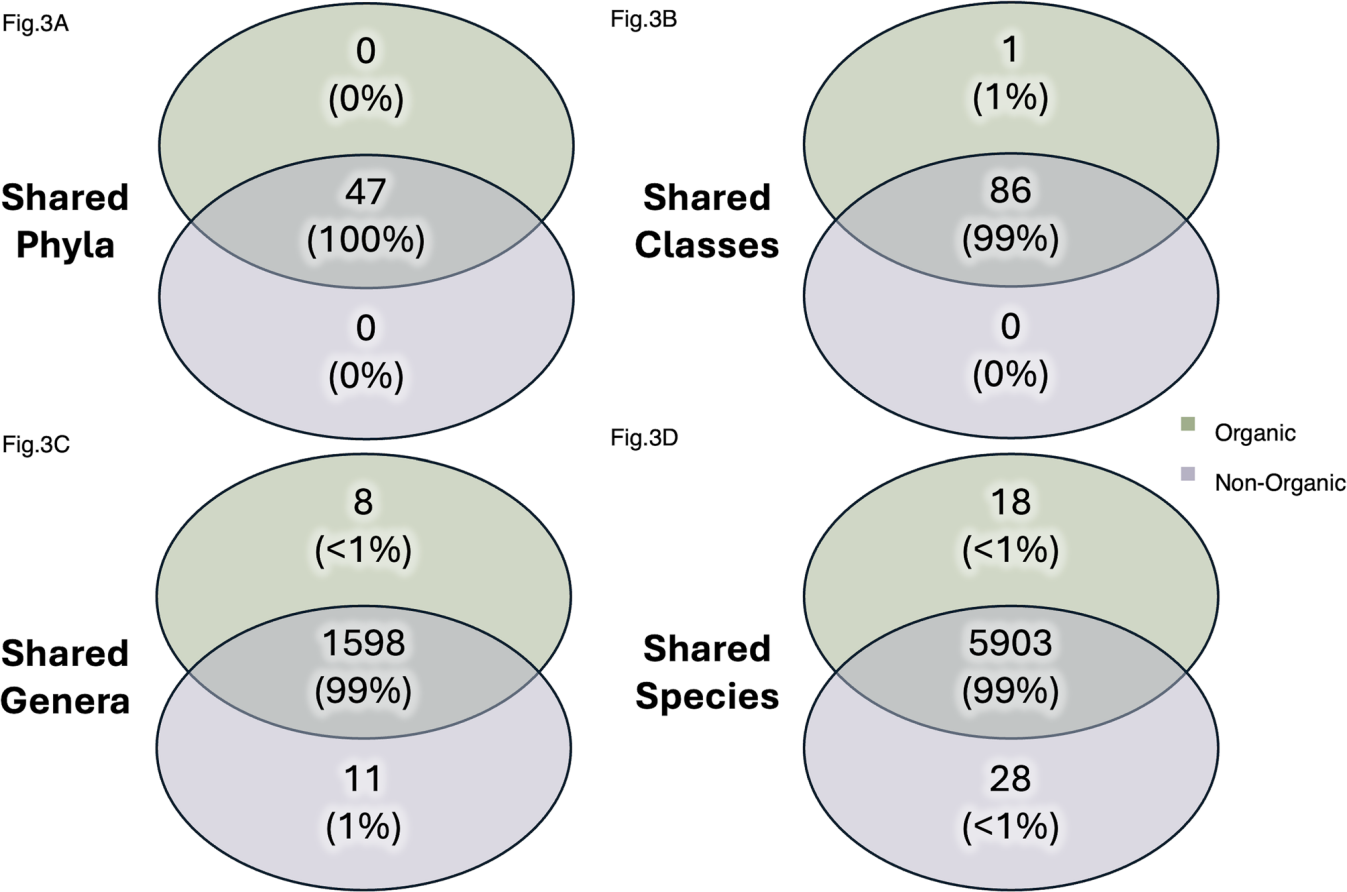
Shared bacterial taxonomy by cropping system. **(A)** Shared number of unique phyla, **(B)** Shared number of unique classes, **(C)** Shared number of unique genera, and **(D)** Shared number of unique species between non-organic (purple) and organic (green) cropping system soils.

Our analyses suggest that organic (O) system soils had higher microbial diversity and bacterial abundance relative to the non-organic (NO) system (Figs. 1 and 2A). Microbial diversity (Shannon’s) was significantly (Wilcoxon Test: *P* = 0.002) higher in the organic system compared to the non-organic system overall. These differences were statistically significant 1-day post-harvest (Wilcoxon Test: *P* = 0.03) but declined in significance by 12-days post-harvest (Wilcoxon Test: *P* = 0.11; Fig. 1), likely because disturbance from harvesting increased variation across samples temporarily. These patterns remained consistent for non-taxonomic (unclassified) and species-level data but were insignificant at higher levels of taxonomy. There were no significant differences in observed microbial richness between organic and non-organic systems, as confirmed by the similarities in total unique taxonomy (phyla: NO:47, O:47; classes: NO:86, O:87; genera: NO:1,609, O:1,606, and species: NO:5,931, O:5,921, Fig. 3).

Overall, there were no significant differences in ARG (Shannon’s) diversity or observed richness within different cropping systems (Supplemental Fig. S1). However, both (Shannon’s) diversity and observed richness for the resistome within organic cropping systems were significantly higher 1-day post-harvest compared to 12-days post-harvest (Supplemental Fig. S2).

### Taxonomy & AMR genes - normalized and relative abundance

The abundance of microbial phyla and classes was significantly different between non-organic and organic systems (PERMANOVA: *F* = 10.66, *P* = 0.001; *F* = 10.75, *P* = 0.001). Overall, the CSS-normalized abundance of the top phyla was higher in the organic system than the non-organic system (Fig. 2A). There were differences in the relative abundance of top phyla (from the top 0.5% and 0.1%) in cropping system microbiomes, with organic (Top 5 phyla: Actinobacteria, Proteobacteria, Planctomycetes, Firmicutes, Acidobacteria) having significantly (PERMANOVA: *F* = 16.58, *P* = 0.001) increased abundances of Actinobacteria compared to non-organic (Top 5 phyla: Proteobacteria, Actinobacteria, Planctomycetes, Firmicutes, and Bacteroidetes), which was significantly (PERMANOVA: *F* = 8.75, *P* = 0.003) more abundant in Proteobacteria (Fig. 2B, Fig. S3A). There also were significant differences in the majority of the (top 0.5%) most abundant bacterial species between non-organic and organic systems (PERMANOVA: *P* < 0.005, Fig. S3B).

**Fig. 4:**
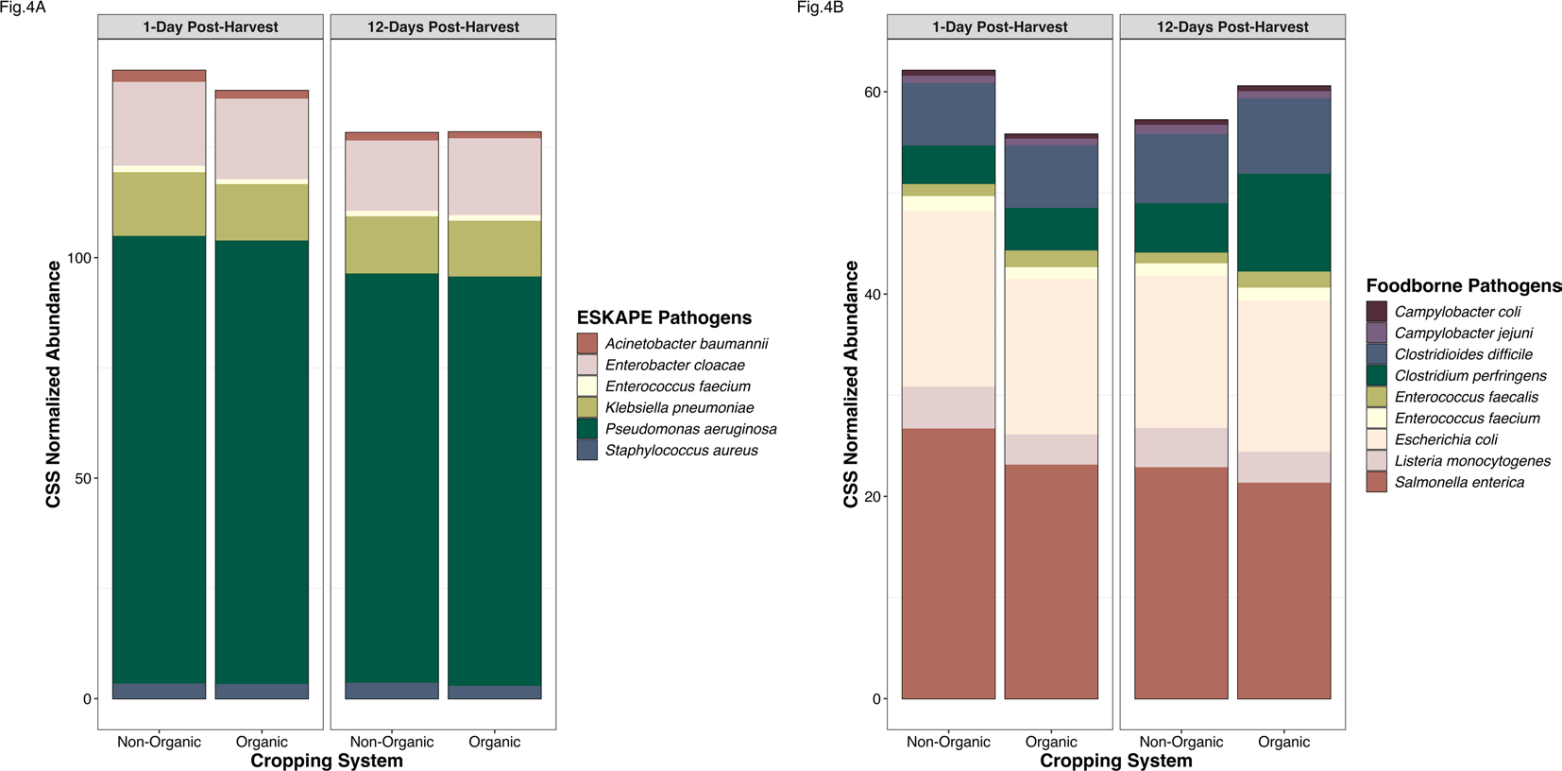
Pathogen abundance by cropping system. **(A)** Cumulative sum scaled (CSS) normalized abundance of WHO ESKAPE pathogens in non-organic and organic cropping system soils 1- and 12- days post- corn harvest and **(B)** CSS normalized abundance of common foodborne pathogens in non-organic and organic cropping system soils 1- and 12- days post- corn harvest.

To understand the impact of these soil management practices on pathogens, we subset the data to include WHO ESKAPE pathogens (0.031% of sequencing data) and a set of common foodborne pathogens (0.014% of sequencing data), both of which were present at very low abundances. The abundance of foodborne pathogens, but not ESKAPE pathogens, differed significantly between cropping systems across time (PERMANOVA: *F* = 2.123, *P* = 0.004). In the pathogen data subsets, ESKAPE and foodborne pathogens were more abundant in the non-organic soil 1-day post-harvest (Fig. 4A and B). However, 12-days post-harvest, foodborne pathogens were more abundant in the organic system and there was no significant difference in abundance of ESKAPE pathogens between systems (Fig. 4A and B), regardless of sampling time (PERMANOVA: *F* = 1.825, *P* = 0.154).

These results indicate that organic practices increased overall bacterial community abundance but did not increase the abundance of pathogens in most cases. Although harvest practices influenced temporal variation in these patterns, the use of composted poultry manure and organic soil management practices appeared to reduce pathogens in newly disturbed soil (1-day post-harvest), whereas non-organic soil management practices appeared to support higher levels of pathogens overall.

**Fig. 5:**
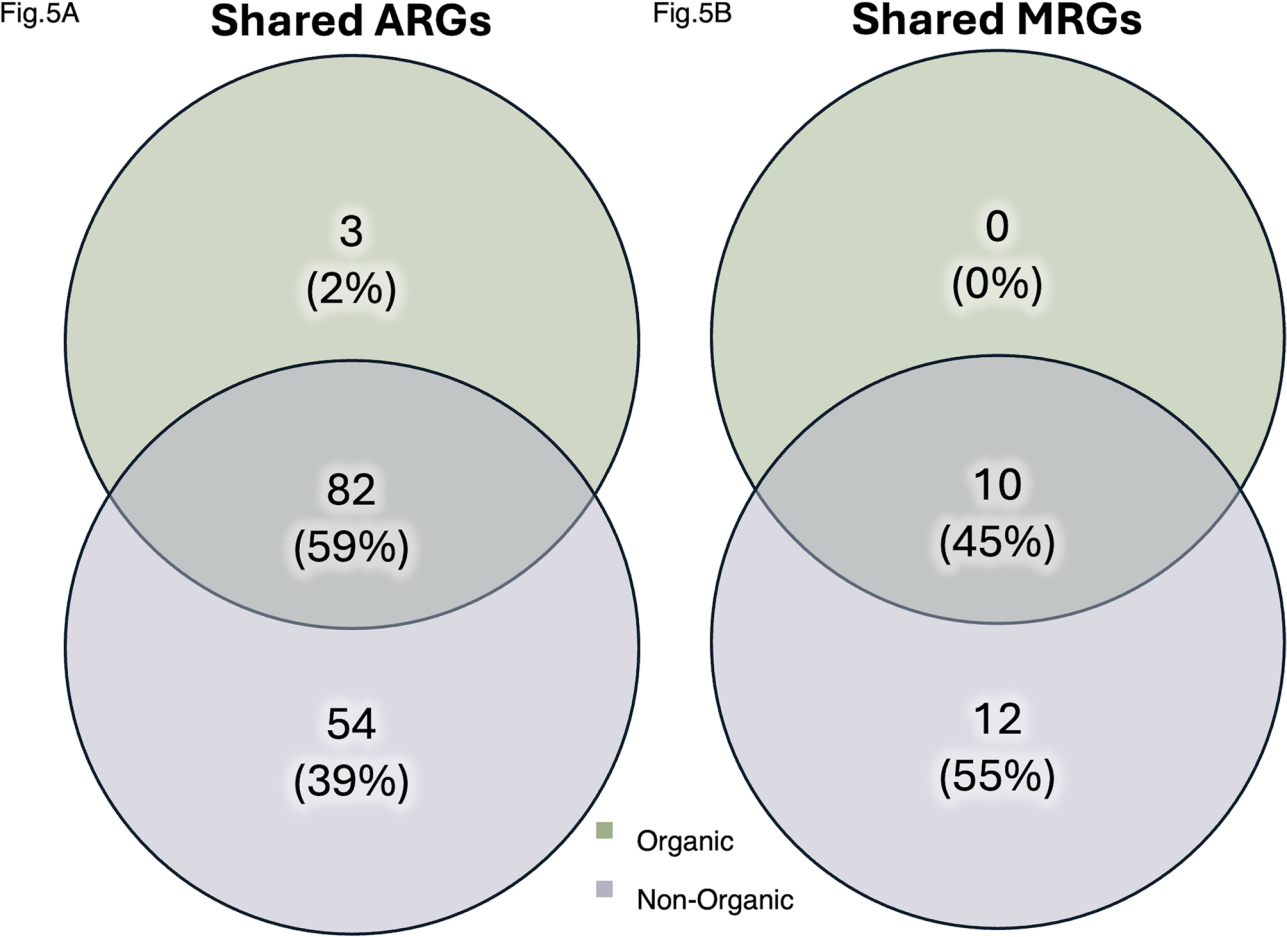
Shared resistance genes by cropping system. **(A)** The number of unique antibiotic-resistant genes (ARGs) shared between non-organic (purple) and organic (green) cropping system soils. **(B)** The number of unique metal resistant genes (MRGs) shared between between non-organic (purple) and organic (green) cropping system soils.

Our total resistance data were composed of 66.95% antimicrobial resistance, 19.4% multi-compound resistance, 11.72% metal resistance, and 1.87% biocide resistance. Overall, more unique ARGs and MRGs were identified in the non-organic samples (ARG = 136, MRG = 22) than in the organic samples (ARG = 85, MRG = 10). The majority of unique ARGs (59%, *n* = 82) and MRGs (45%, *n* = 10) were shared between both cropping systems. However, the non-organic system had a greater number of uniquely identified ARGs (39%, *n* = 54) and MRGs (55%, *n* = 12), compared to the organic system (ARGs: 2%, *n* = 3; MRGs: 0%, *n* = 0) (Fig. 5). Similarly, more unique resistance mechanisms were identified in the non-organic system (MR = 12, AMR = 60) than in the organic system (MR = 10, AMR = 48).

**Fig. 6:**
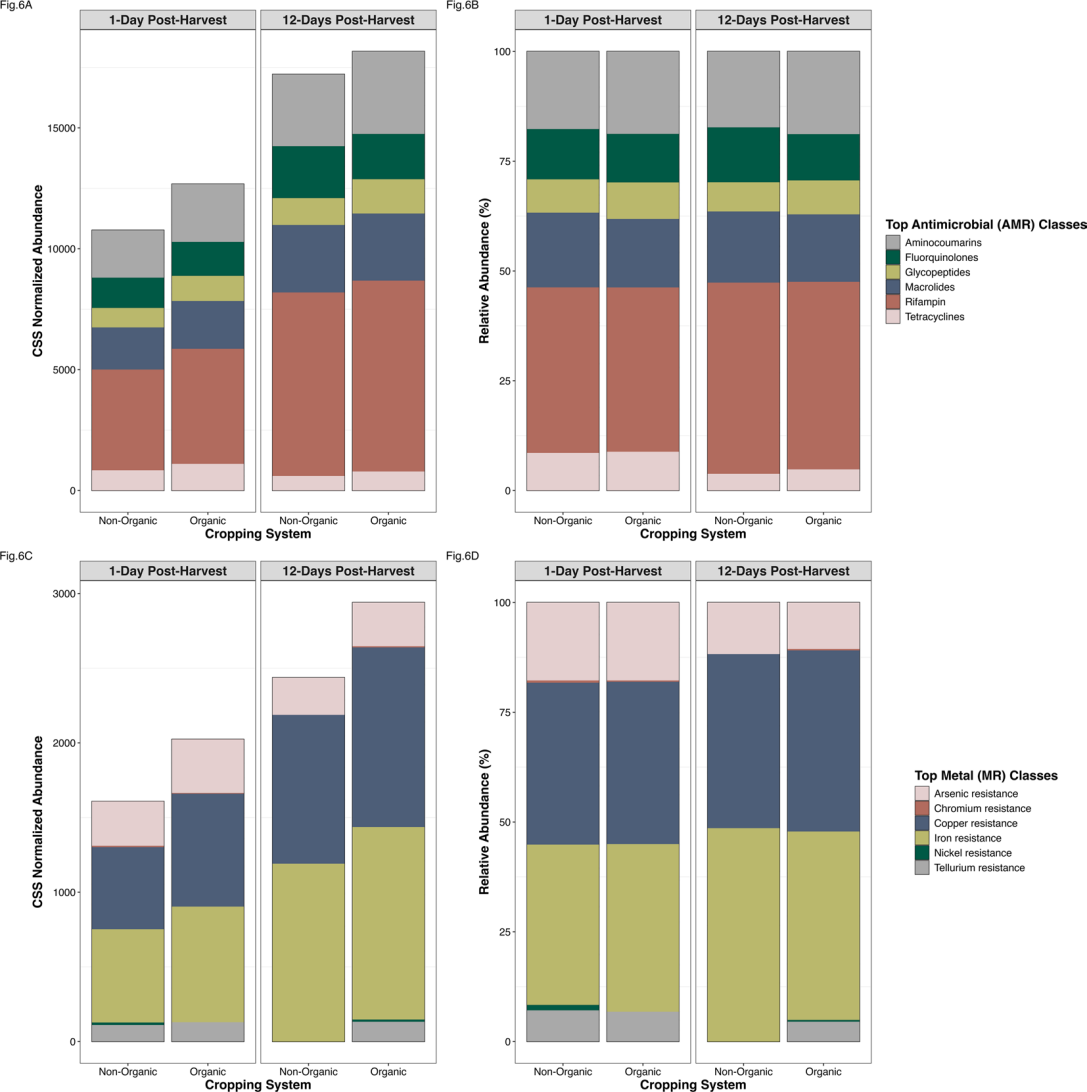
Top resistance classes of the resistome. Resistance classes that represent the top 5% of all antimicrobial resistance (AMR) or metal resistance (MR) class counts in non-organic or organic cropping system soils by 1- or 12- days post- corn harvest. **(A)** Cumulative sum scaled (CSS) normalized AMR class abundance. **(B)** Relative AMR class abundance. **(C)** CSS normalized MR class abundance. **(D)** Relative MR class abundance.

Although the non-organic samples had a broader range of unique ARGs and MRGs, these genes were present at lower abundances compared to the organic group (Fig. S4A & S4B). These higher normalized abundances of resistance genes in the organic system are reiterated within the top abundant ARG and MRG classes (Fig. 6). Additionally, the most abundant (from the top 0.5%) ARGs and antibiotic resistance mechanisms differed in abundance between organic and non-organic systems (Fig. S5A and S5B). Taken together, these results indicate that the organic systems, amended with composted poultry manure, were associated with higher abundance of both ARGs and MRGs, but a narrow range of unique resistance genes and mechanisms.

**Fig. 7:**
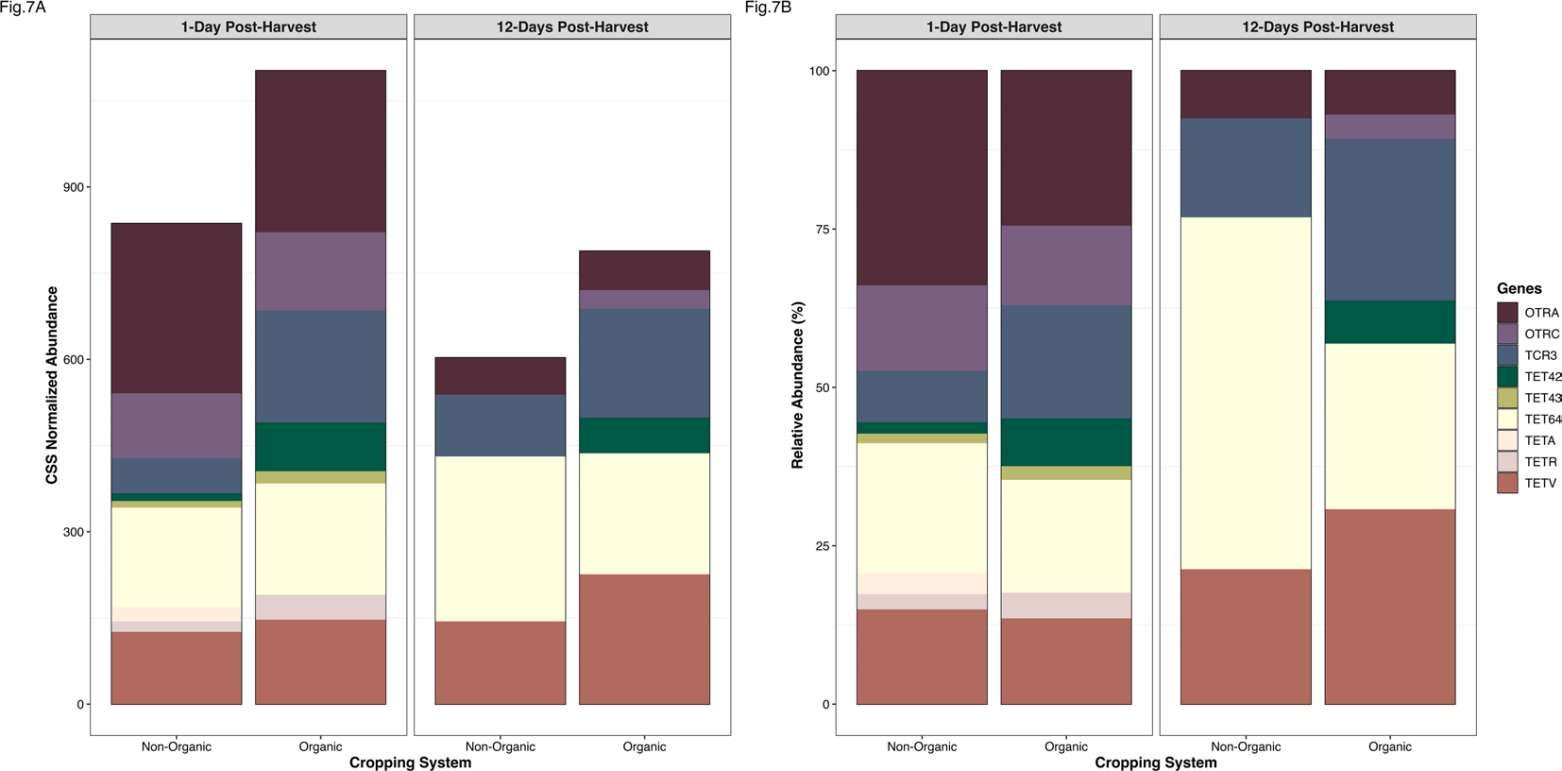
Abundance of tetracycline and oxytetracycline genes. These abundances are shown in non-organic and organic cropping system soils 1- day and 12-days post-harvest. **(A)** Cumulative sum scaled (CSS) normalized abundance of *tet* genes **(B)** Relative abundance of *tet* genes.

We also found a higher abundance of ARGs 12-days post-harvest in both cropping systems—the opposite pattern observed for bacterial and pathogen abundance. The relative abundance of specific AMR classes was similar between non-organic and organic systems, but tetracyclines were significantly more abundant in the organic system over time (PERMANOVA: *F* = 2.637, *P* = 0.053; Fig. 6A and B). There was also a decline in tetracycline ARGs in both systems over the 12-day post-harvest period. Overall, *tet*64, *otr*A (oxytetracycline resistance), and *tet*V were the most abundant tetracycline resistance genes identified. Notably, *tet*A was detected only in the non-organic system 1-day post-harvest, and at lower abundance than other *tet* genes. The abundance of individual *tet* genes varied by cropping system and time point, but levels were too low to detect statistically significant differences between groups (Fig. 7).

Patterns of MRG abundance across collection time points and cropping systems mirrored those of ARGs (Fig. 6C and D). Higher abundances of nickel and chromium resistance genes were observed in the non-organic system, and interestingly, zinc resistance genes were detected only in the non-organic system 1-day post-harvest (Fig. S4B). However, none of these differences were statistically significant.

### Microbiome and resistome - composition similarities

**Fig. 8:**
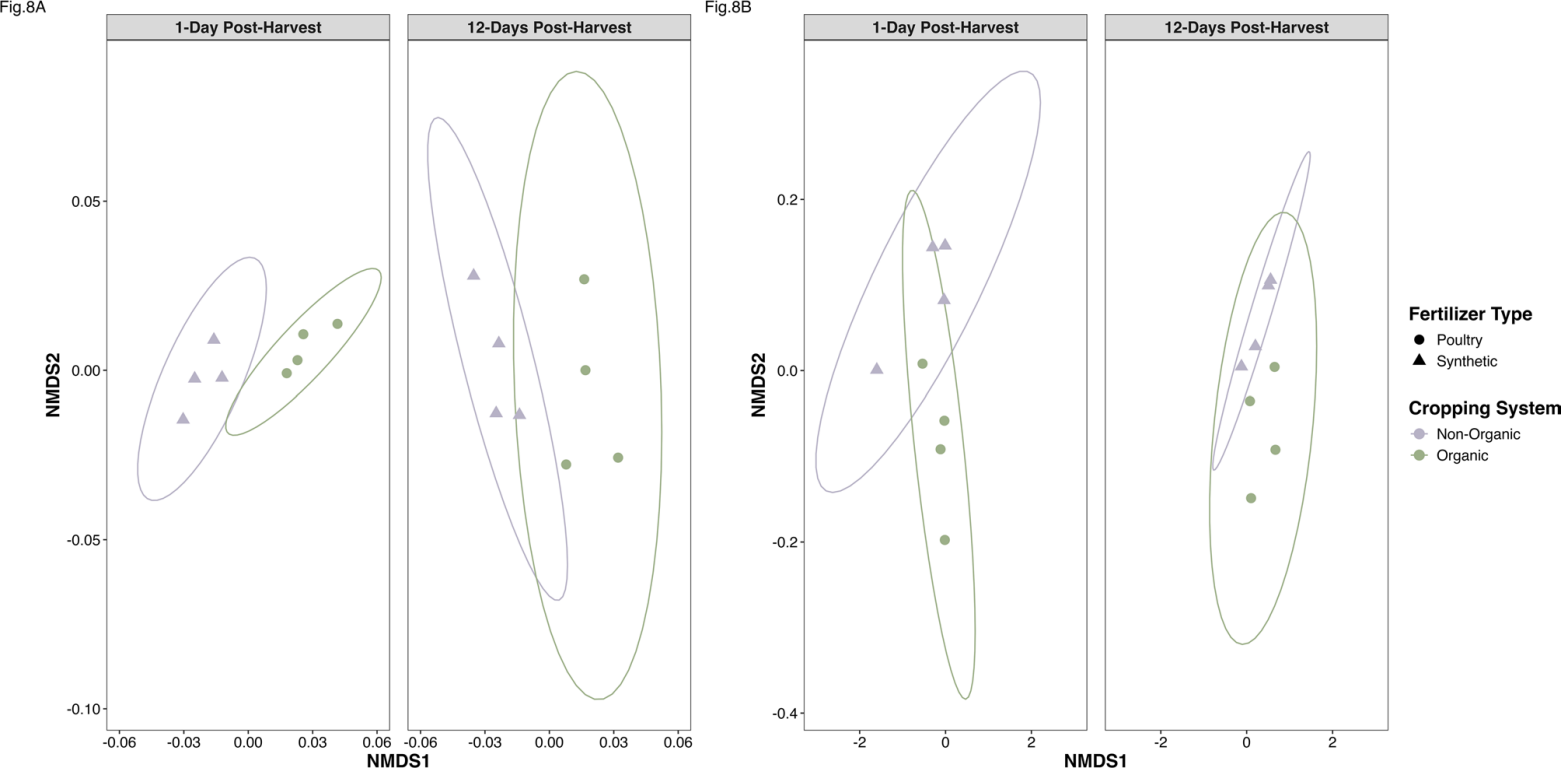
Beta diversity of the microbiome and resistome. **(A)** Non-metric multidimensional scaling (NMDS) of bacterial class by cropping systems (non-organic = purple, organic = green, synthetic = triangle, poultry manure = circle) across 1- to 12-days post-harvest (Stress = 0.095, Non-metric fit *R*^*2*^ = 0.993.) **(B)** NMDS of AMR group by cropping systems (non-organic = purple, organic = green, synthetic = triangle, poultry manure = circle) across 1- to 12-days post-harvest (Stress = 0.036, Non-metric fit *R*^*2*^ = 0.999).

The composition of bacterial classes differed significantly by cropping system (PERMANOVA: *F* = 3.693, *P* = 0.006) and showed varying levels of convergence over time (PERMANOVA: *F* = 2.441, *P* = 0.043). These bacterial classes were most distinct when considering the interaction between cropping system and collection time (PERMANOVA: *F* = 10.658, *P* = 0.001; NMDS: Stress = 0.095, Non-metric fit *R*^*2*^ = 0.993; Fig. 8A). This pattern remained consistent across taxonomic levels (Supplementary Fig. S6A & S6B).

The ARG composition varied more 1-day post-harvest compared to 12-days post-harvest. Both systems were more similar when grouped by AMR class (Stress = 0.097, *R*^*2*^ = 0.991; Supplemental Fig. S7A) and AMR mechanism (Stress = 0.057, Non-metric fit *R*^*2*^ = 0.997; Supplemental Fig. S7B) than by AMR group (Stress = 0.036, Non-metric fit *R*^*2*^ = 0.999, Fig. 8B). These results suggest that the organic cropping systems amended with composted poultry manure were characterized by soil microbial communities with higher abundances and more diverse bacteria as well as more abundant — but less diverse — ARGs and MRGs. Thus, in this system, higher microbial diversity is associated with more abundant but less varied resistome profiles.

### Resistome risks & mobile genetic elements

**Fig. 9:**
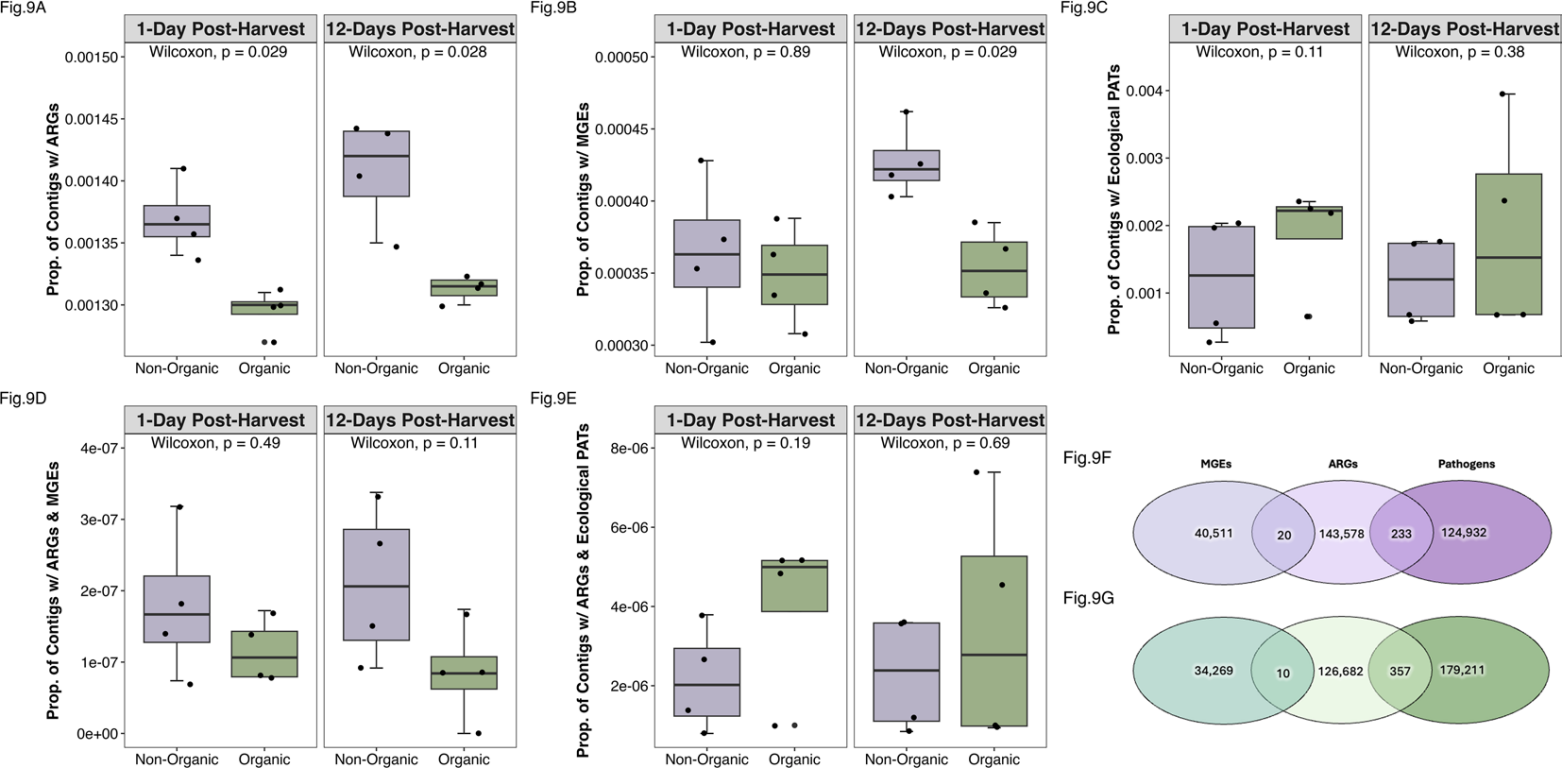
Illustrating ecological risk score components through the relative contributions of antimicrobial resistance genes (ARGs), mobile genetic elements (MGEs), and ecological (non-ESKAPE) pathogens (PATs) in non-organic and organic cropping systems. **(A)** Proportion of contigs containing ARGs (nARGs/nContigs). **(B)** Proportion of contigs containing MGEs (nMGEs/nContigs). **(C)** Proportion of contigs aligning with ecological PATs (nPATs/nContigs). **(D)** Proportion of contigs containing ARGs and aligning with ecological PATs (nARGs + PATs/nContigs). **(E)** Proportion of contigs containing ARGs and MGEs (nARGs + MGEs/nContigs). **(F)** Contigs from non-organic (purple) cropping system soil with ARG, MGE, and ecological PAT alignment, and combinations of both. **9G)** Contigs from organic (green) cropping system soil with ARG, MGE, and ecological PAT alignment, and combinations of both. There were no contigs containing ARGs, MGEs, and PATs alignment altogether.

We also investigated whether soil management practices impact the risks of transmitting drug-resistant pathogens. Here, sequencing data were assembled to quantify the number and proximity of ARGs, MGEs *and* pathogen sequences, as indicated by their co-presence within the same contigs. Pathogens were categorized into human health pathogens (ESKAPE pathogens) and ecological pathogens (non-ESKAPE pathogens). There were more contigs aligning with ESKAPE pathogens in the non-organic system (6 contigs) compared to the organic system (3 contigs). In contrast, the organic system (179,211 contigs) had more contigs aligning with non-ESKAPE pathogens relative to the non-organic system (124,932 contigs; Fig. 9).

Importantly, no contigs were identified that contained combinations of MGEs, ARGs, and ecological (non-ESKAPE) pathogen alignment all together. There also were no contigs that contained MGEs with Class 1 ARGs or MGEs with ESKAPE pathogens. These findings suggest a limited potential for horizontal gene transfer involving high-risk resistance genes and human pathogens, as evidenced by lower human health risk scores in both cropping systems compared to ecological risk scores.

**Fig. 10:**
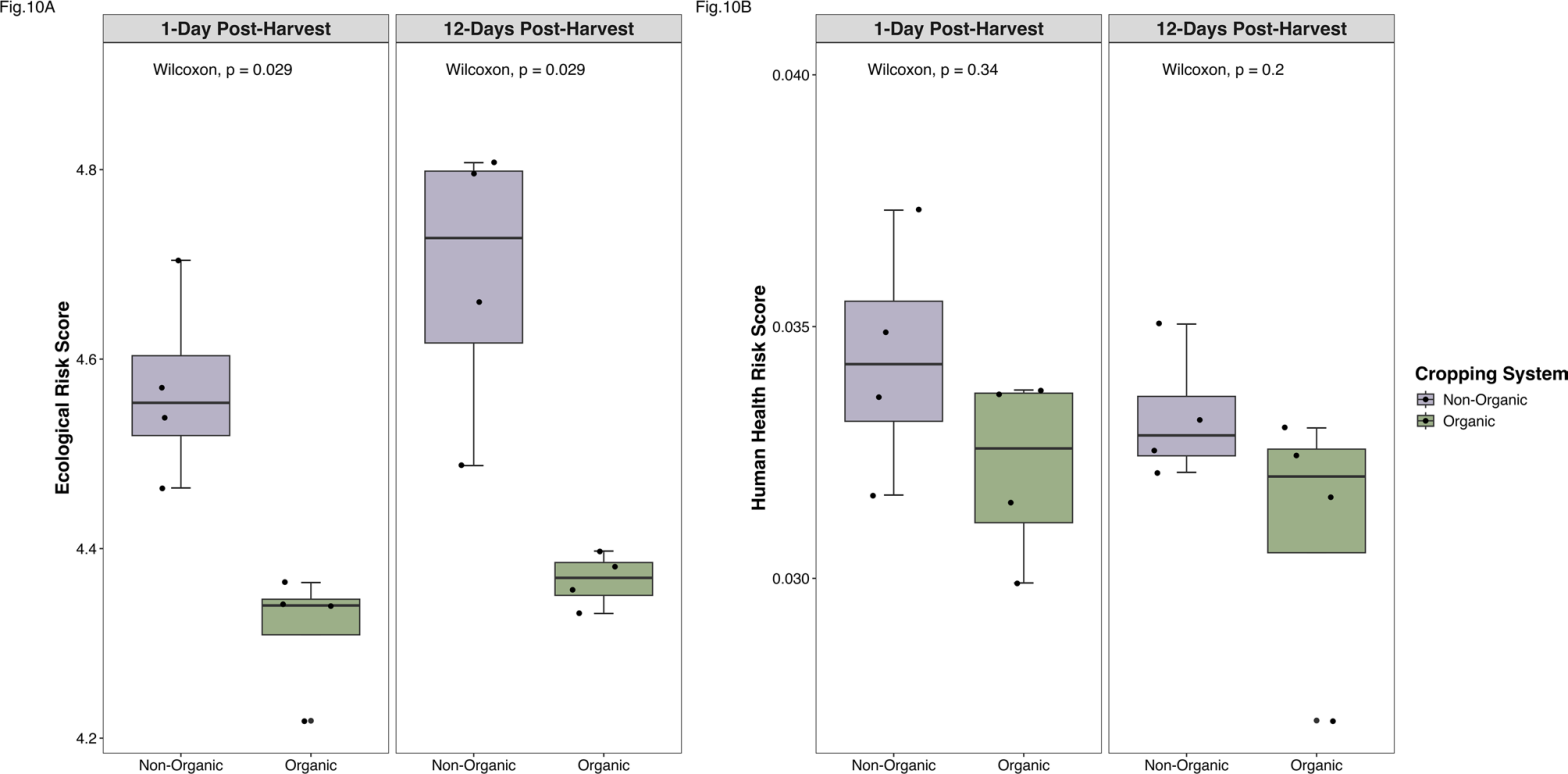
Ecological and human health resistome risk scores. **(A)** Ecological risk scores were significantly different for non-organic (purple) and organic (green) cropping system soils by 1-day (Wilcoxon test: *P* = 0.03) and 12-days (Wilcoxon test: *P* = 0.03) post-harvest. **(B)** Human health risk scores were statistically insignificant for non-organic (purple) and organic (green) cropping system soils by 1-day (Wilcoxon test: *P* = 0.34) and 12- days post-harvest (Wilcoxon test: *P* = 0.20).

A high risk score represents an increased likelihood of drug-resistant pathogens spreading through indirect (environment-host) or direct (host-host) transmission pathways. Our risk score analysis suggests that indirect transmission risks (ecological risk scores), but not direct transmission risks (human health risk scores), were significantly higher (Wilcoxon Test: *P* = 0.03) in the non-organic system compared to the organic system (Fig. 10), contrasting with the ARG and MRG abundance trends discussed above (Fig. 6).

**Fig. 11:**
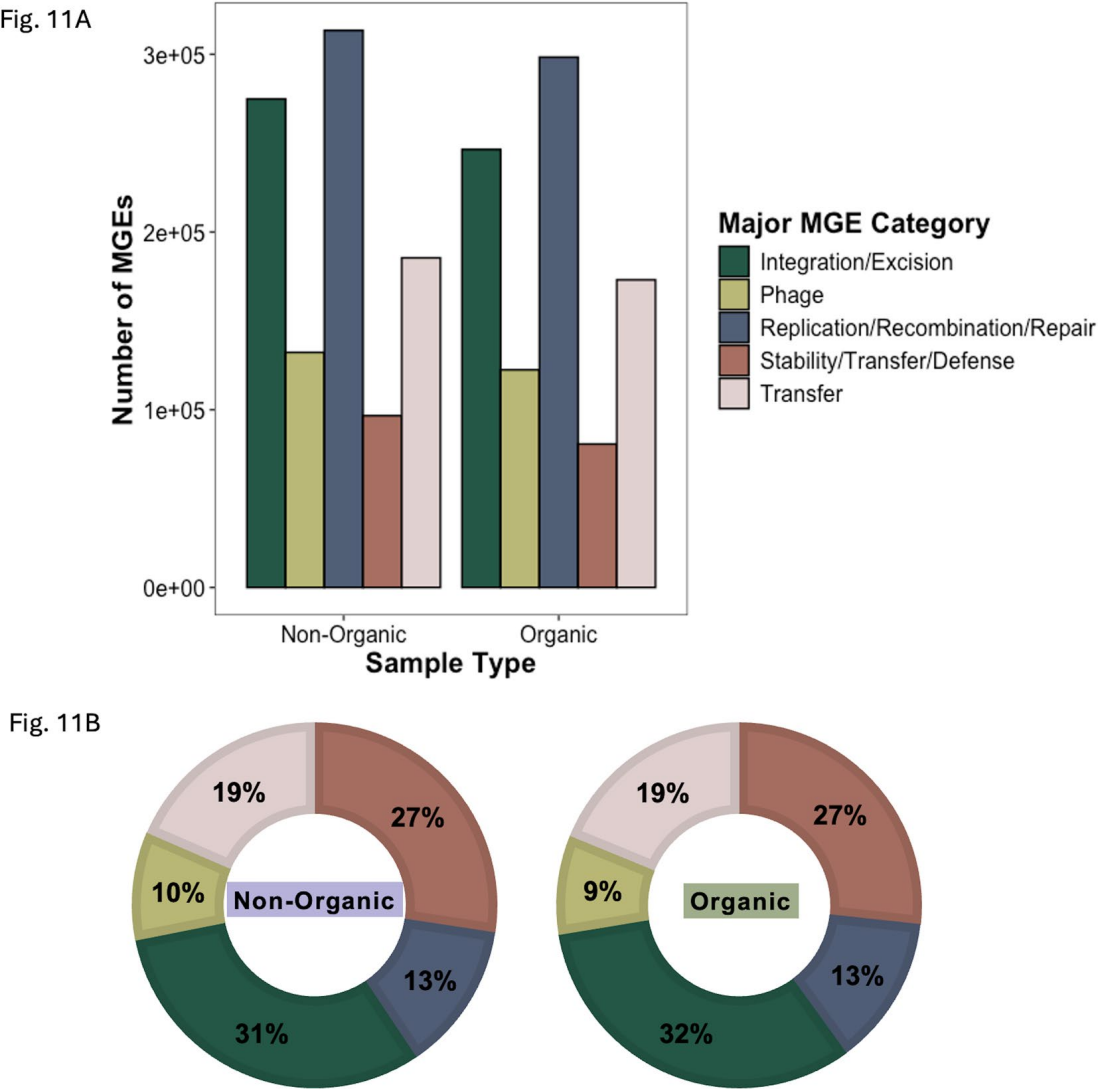
Mobile genetic elements by functional categories. (**A)** The bar plots show the number of mobile genetic elements (nMGEs) identified within contigs classified by major functional categories for non-organic and organic cropping system soils. **(B)** The donut plots show the proportions (nMGEs/nContigs) of MGE major functional categories by cropping system.

Unlike the analyses above, which focus solely on abundance and diversity of ARGs, these risk scores assess transmission potential by including the presence of mobile genetic elements in the calculation. There were more contigs containing individual MGEs (NO = 40,511 O = 34,269) as well as more contigs containing combinations of both ARGs and MGEs (NO = 20, O = 10) within the non-organic system compared to the organic system (Fig. 9A,D,F,G). Although the composition of MGE functions — integration/excision, phage, replication/recombination/repair, stability/transfer/defense, and transfer — were similar between each system, the non-organic system exhibited elevated levels of MGEs across all functional categories compared to the organic system (Fig. 11), especially at 12-days post-harvest (Fig. S8).

A total of 25 unique integrases and 29 unique transposases were identified across both cropping systems. Two transposases, *tnp*A (NO = 15,423 counts, O = 14,249 counts) and *tnp*M (NO = 12,276 counts, O = 11,647 counts), were among the top 10 most abundant MGEs in both systems (Fig. S9). Notably, *int*1 and *int*2 were also detected, but at lower abundances than the transposases (Fig. S10). Overall, the non-organic system exhibited a higher abundance of both transposases and integrases compared to the organic system, although these differences were not statistically significant (Fig. S11). Together, the risk score analyses contribute to a more robust assessment of resistome risk, emphasizing the importance of MGE and resistance gene co-presence rather than relying solely on ARG and MRG diversity and abundance as indicators.

## Discussion

Our large-scale field study offers insights into how soil management practices shift soil microbial diversity and the risks of transmitting antimicrobial resistance genes and pathogens. Non-organic systems in our study were associated with high risks of transmitting both environmental pathogens and antimicrobial resistance genes (ARGs). These risks were driven primarily by the higher abundance of mobile genetic elements (MGEs), rather than simply the abundance of ARGs. In fact, ARG abundance was lower in non-organic soils compared to organic soils. More specifically, non-organic soil management practices, that used synthetic fertilizers and biocides, had a higher abundance of contigs with MGEs and double the number of contigs containing both MGEs and ARGs. Additionally, non-organic soils contained a broader array of unique resistance genes and mechanisms. This diversity, combined with high MGE levels, points to a more complex and potentially mobile resistome.

In contrast, organic systems were characterized by higher total abundances of ARGs that consisted of a narrow range of unique resistance genes and mechanisms, but low levels of MGEs. Macrolide, fluoroquinolone, and tetracycline resistance genes were abundant in the organic system, consistent with other poultry manure studies and the substantial use of these antimicrobials in poultry farming for disease prevention and treament^22,76^. The resistance traits in these organic systems appear less diverse, with fewer mobile genetic elements to facilitate horizontal gene transfer. Moreover, this suggests that although organic soil management practices, including the use of poultry manure as fertilizer, may enrich the resistome, they do so in a more constrained and potentially less transmissible form. As a result, organic practices may lower the risks associated with spreading pathogens and antimicrobial resistance, despite higher overall abundance of ARGs.

While these patterns are interesting, pinpointing the underlying ecological and evolutionary mechanisms driving these emergent patterns is crucial. This study points to at least two potential mechanisms that may explain the observed patterns in the abundance, diversity, and transmission of pathogens and antimicrobial resistance genes. First, the non-organic system was characterized by less abundant and less diverse soil microbial communities, in direct contrast to organic systems amended with poultry manure. Thus, in this system, higher microbial diversity was associated with more abundant but less diverse resistome profiles and lower overall transmission risks. These results could arise due to competitive interactions among microbes and the ostensible energetic costs associated with building and maintaining ARGs or metal resistance genes^56,77,78^. Of course, a combination of laboratory and field studies within complex microbial communities is needed to address these intriguing interactions. While such experiments are beyond the scope of this study, improving soil microbial diversity to reduce pathogens and AMR genes within environmental reservoirs could present a win-win management solution.

Second, our results suggest potential for co-selection among antimicrobial resistance genes, mobile genetic elements, and metal resistance genes (MRGs)^12,27^. Specifically, across the organic cropping system, MRGs were positively correlated with ARGs but negatively correlated with mobile genetic elements. These patterns may reflect selective pressures that favor ARGs and MRGs together, while potentially disfavoring MGEs, raising the possibility of competition or trade-offs among these genes. The functional classes of MGEs were similar across systems and the major functional categories were integration/excision and replication/recombination/repair. However, 55% of these genes were unique to non-organic systems, suggesting distinct transmission pathways or mechanisms promoting the functional diversity of MGEs. Potential explanations for these patterns may be that metal supplementation in poultry feed is a common practice and composting poultry manure can concentrate these metals, leading to lower functional diversity and sustained heavy metal accumulation in soils amended with manure fertilizers^12,22,27,79^.

In addition to the broad implications of heavy metal concentration in the environment, these metals may co-select for specific ARGs, contributing to a narrow resistome profile. In our samples, Fe and Cu MRGs were the most abundant. Cu resistance is known to be strongly correlated with the prevalence of macrolide, fluoroquinolone, tetracycline, beta-lactamase, and aminoglycoside ARGs (*erm*C, *qnr*S, *tet*M, *bla*_CTX−M_, and *aac(6’)-Ib*-cr) in soils amended with poultry litter^12,24^. Selective pressures from residual metals and the resultant co-selection among ARGs and MRGs may explain why, in our study, ARGs from these resistance classes were common. The complex patterns we observed here warrant future investigations as they involve trade-offs among management goals and the associated hazards and long-term risks. For instance, while poultry manure used as fertilizer in combination with organic practices could reduce the risks associated with the spread of drug-resistant pathogens, including foodborne pathogens of public health concern, they could also inadvertently concentrate heavy metals. Perhaps more importantly, investigating interactions among these two major public health concerns may lead to novel interventions obscured by studying either of these issues alone.

Do the patterns observed here represent short-term perturbations or long-term equilibria? Unfortunately, the answer to this question is beyond the scope of the current study. Here, we focused on fertilizer amendment patterns associated with a subset of key management practices predicted to alter the distribution of pathogens and the resistome. These snapshot studies offer compelling but incomplete insight, and more studies are needed to address temporal variation. To date, most studies examining the effects of poultry manure fertilizers use single snapshots, due in large part to logistical constraints and the costs of deep sequencing^80–82^. These short-term studies are exceptionally useful in guiding more long-term studies. Indeed, the results from this current study informed our efforts to investigate fine-scale variation in microbial and resistome dynamics across the entire growing season (Nickodem et al., *in prep*).

Notably, this current study uncovered another puzzling pattern that warrants further investigation. Surprisingly, we found that genes for rifampin, aminocoumarin, and glycopeptide resistance were more abundant than expected, because these ARGs are commonly identified in soils that are in close proximity to cattle^83^. One possible explanation is that our sites are embedded within a larger, ongoing study that includes multiple long term soil management practices, including the application of dairy manure. Thus, we cannot rule out the possibility that the focal sites received some runoff from these sites or that equipment-related transfer contributed to these results. In many ways cross-contamination among treatments would provide a simple explanation. Yet, this seems unlikely given the broad patterns and differences among treatments receiving dairy manure (Nickodem et al., *in prep*).

To understand the dynamics of pathogens and antimicrobial resistance, it is essential to examine how these elements interact within the complex microbial communities where they reside. This goal requires moving beyond the lab to field studies, which leads to a suite of trade-offs among study designs. Our current experimental design takes a top-down approach reflecting industry-standard cropping system techniques and cannot, as of yet, isolate specific management practices^13^ .

This experimental design is notably different from most studies focused on poultry manure fertilizers. These studies often compare poultry manure to fertilizer-free controls. While this simplified design can isolate specific interactions, it employs a scenario that is rarely used in real-world farming. The design used by WICST was intentionally developed with the goal to explore alternative land management strategies aimed at improving crop productivity. For the purposes of our study, we can leverage these realistic farming practices to ask questions that would not be feasible with a full factorial design. For instance, implementing a full factorial design across four randomized blocks would require at least 16 additional plots and 5.44 hectares of land, an impractical expansion given time, space, and the cost of deep metagenomic sequencing. Perhaps more importantly, while such comparisons might be interesting from a pure science perspective, they would not advance our central goal of providing practical guidance for land managers, policymakers, or stakeholders. Thus, while we fully acknowledge the limitations of the current experimental design, the wealth of information gained from this study and others at WICST highlight its unique and unparalleled contributions to both basic and applied science.

Together, these findings underscore the value of risk score analyses, as most studies examining environmental reservoirs of antimicrobial resistance are general and, in particular, they tend to use ARG abundance as a proxy for AMR risk when determining the impact of poultry manure^12,22–25^. Our results contribute to a growing body of evidence suggesting that MGEs may be more informative indicators to understand not just risks but also the mechanisms driving the emergence and spread of AMR. By incorporating mobile genetic elements and risk score analyses, we gain a more nuanced understanding of transmission potential, highlighting that gene mobility and ecological context are critical to evaluating AMR risks in agricultural environments. Future studies investigating how these patterns impact evolutionary dynamics may be particularly insightful. For instance, the combination of diverse resistance mechanisms and higher levels of mobile elements in the non-organic systems could create a more flexible genetic landscape, enabling pathogens to rapidly adapt to selective pressures, including antimicrobial use. Over time, this could lead to the emergence of novel resistance profiles and more persistent AMR threats in agricultural soils. Unraveling these eco-evolutionary mechanisms could reveal new opportunities to leverage soil management practices that reduce the emergence and spread of AMR in environmental reservoirs.

## Supplementary Information

Below is the link to the electronic supplementary material.

Supplementary Material 1

## Data Availability

The datasets generated and analyzed during the current study are available in the NCBI Sequence Read Archive (SRA) database under the accession number PRJNA1277182.

## References

[1] Llor, C. & Bjerrum, L. Antimicrobial resistance: risk associated with antibiotic overuse and initiatives to reduce the problem. *Ther. Adv. Drug Saf.* **5**, 229–241 (2014). 25436105 10.1177/2042098614554919 PMC4232501

[2] Van Boeckel, T. P. et al. Global trends in antimicrobial use in food animals. *Proc. Natl. Acad. Sci. U S A*. **112**, 5649–5654 (2015). 25792457 10.1073/pnas.1503141112 PMC4426470

[3] Martin, M. J., Thottathil, S. E. & Newman, T. B. Antibiotics overuse in animal agriculture: A call to action for health care providers. *Am. J. Public. Health*. **105**, 2409–2410 (2015). 26469675 10.2105/AJPH.2015.302870 PMC4638249

[4] NARMS. 2019 NARMS Update: Integrated Report (2019).

[5] Vidovic, N. & Vidovic, S. Antimicrobial resistance and food animals: influence of livestock environment on the emergence and dissemination of antimicrobial resistance. *Antibiotics (Basel)* **9** (2020). 10.3390/antibiotics9020052 PMC7168261 32023977

[6] Cameron, A. & McAllister, T. A. Antimicrobial usage and resistance in beef production. *J. Anim. Sci. Biotechnol.* **7**, 68 (2016). 27999667 10.1186/s40104-016-0127-3 PMC5154118

[7] Ager, E. O., Carvalho, T., Silva, E. M., Ricke, S. C. & Hite, J. L. Global trends in antimicrobial resistance on organic and conventional farms. *Sci. Rep.* **13**, 22608 (2023). 38114527 10.1038/s41598-023-47862-7 PMC10730711

[8] Ng, C. et al. Characterization of metagenomes in urban aquatic compartments reveals high prevalence of clinically relevant antibiotic resistance genes in wastewaters. *Front Microbiol* **8** (2017). 10.3389/fmicb.2017.02200 PMC5696577 29201017

[9] Ghaly, T. M. & Gillings, M. R. New perspectives on mobile genetic elements: a paradigm shift for managing the antibiotic resistance crisis. *Philos. Trans. R Soc. Lond. B Biol. Sci.* **377**, 20200462 (2021). 34839710 10.1098/rstb.2020.0462 PMC8628067

[10] Thanner & Sophie Drissner David & Walsh Fiona. *Antimicrob. Resist. Agric. MBio*. **7**10.1128/mbio.02227-15 (2016). 10.1128/mBio.02227-15 PMC4850276 27094336

[11] Lima, T., Domingues, S. & Da Silva, G. J. Manure as a potential hotspot for antibiotic resistance dissemination by horizontal gene transfer events. *Vet Sci* **7** (2020). 10.3390/vetsci7030110 PMC7558842 32823495

[12] Deng, W. et al. Heavy metals, antibiotics and nutrients affect the bacterial community and resistance genes in chicken manure composting and fertilized soil. *J. Environ. Manage.* **257**, 109980 (2020). 31868641 10.1016/j.jenvman.2019.109980

[13] Buta-Hubeny, M. et al. Structure of the manure resistome and the associated mobilome for assessing the risk of antimicrobial resistance transmission to crops. *Sci. Total Environ.* **808**, 9 (2022). 10.1016/j.scitotenv.2021.152144 34864022

[14] Buta, M. et al. Microbial and chemical pollutants on the manure-crops pathway in the perspective of one health holistic approach. *Sci. Total Environ.* **785**, 15 (2021). 10.1016/j.scitotenv.2021.147411 33957582

[15] Yang, X., Hu, H. W., Yang, G. W., Cui, Z. L. & Chen, Y. L. Crop rotational diversity enhances soil Microbiome network complexity and multifunctionality. *Geoderma* **436**, 116562 (2023).

[16] Mo, Y. et al. Agricultural practices influence soil Microbiome assembly and interactions at different depths identified by machine learning. *Res. Square*. 10.21203/rs.3.rs-3959167/v1 (2024). 10.1038/s42003-024-07059-8 PMC11489707 39424928

[17] Wang, H. et al. Long-term herbicide residues affect soil multifunctionality and the soil microbial community. *Ecotoxicol. Environ. Saf.* **283**, 116783 (2024). 39067076 10.1016/j.ecoenv.2024.116783

[18] Cui, E., Wu, Y., Zuo, Y. & Chen, H. Effect of different biochars on antibiotic resistance genes and bacterial community during chicken manure composting. *Bioresour Technol.* **203**, 11–17 (2016). 26720134 10.1016/j.biortech.2015.12.030

[19] Qiu, X. W., Zhou, G. X., Chen, L. & Wang, H. J. Additive quality influences the reservoir of antibiotic resistance genes during chicken manure composting. *Ecotoxicol. Environ. Saf.* **220**, 8 (2021). 10.1016/j.ecoenv.2021.112413 34139628

[20] Qian, X. et al. Diversity, abundance, and persistence of antibiotic resistance genes in various types of animal manure following industrial composting. *J. Hazard. Mater.* **344**, 716–722 (2018). 29154097 10.1016/j.jhazmat.2017.11.020

[21] Li, J. et al. Long-term manure application increased the levels of antibiotics and antibiotic resistance genes in a greenhouse soil. *Appl. Soil. Ecol.* **121**, 193–200 (2017).

[22] He, L. Y. et al. Dissemination of antibiotic resistance genes in representative broiler feedlots environments: identification of indicator ARGs and correlations with environmental variables. *Environ. Sci. Technol.* **48**, 13120–13129 (2014). 25338275 10.1021/es5041267

[23] Pellegrini, M. C., Okada, E., González Pasayo, R. A. & Ponce, A. G. Prevalence of Escherichia coli strains in horticultural farms from argentina: antibiotic resistance, biofilm formation, and phylogenetic affiliation. *Environ. Sci. Pollut Res. Int.* **29**, 23225–23236 (2022). 34802078 10.1007/s11356-021-17523-1

[24] Wang, L. et al. Macrolide- and quinolone-resistant bacteria and resistance genes as indicators of antibiotic resistance gene contamination in farmland soil with manure application. *Ecol. Indic.* **106**, 105456 (2019).

[25] Wei, Z. et al. Organic fertilizer potentiates the transfer of typical antibiotic resistance gene among special bacterial species. *J. Hazard. Mater.* **435**, 128985 (2022). 35483268 10.1016/j.jhazmat.2022.128985

[26] Tripathi, A. et al. Resistome profiling reveals transmission dynamics of antimicrobial resistance genes from poultry litter to soil and plant. *Environ. Pollut*. **327**, 121517 (2023). 36990341 10.1016/j.envpol.2023.121517

[27] Mazhar, S. H. et al. Co-selection of antibiotic resistance genes, and mobile genetic elements in the presence of heavy metals in poultry farm environments. *Sci. Total Environ.* **755**, 142702 (2021). 33049532 10.1016/j.scitotenv.2020.142702

[28] Ngogang, M. P. et al. Microbial contamination of chicken litter manure and antimicrobial resistance threat in an urban area setting in Cameroon. *Antibiot. (Basel)*. **10**, 20 (2020). 10.3390/antibiotics10010020 PMC7824223 33383622

[29] Chen, Z. & Jiang, X. Microbiological safety of chicken litter or chicken litter-based organic fertilizers: A review. *Agriculture* **4**, 1–29 (2014).

[30] Bonin, N. et al. MEGARes and AMR++, v3.0: an updated comprehensive database of antimicrobial resistance determinants and an improved software pipeline for classification using high-throughput sequencing. *Nucleic Acids Res.* **51**, D744–D752 (2023). 36382407 10.1093/nar/gkac1047 PMC9825433

[31] Doster, E. et al. MEGARes 2.0: a database for classification of antimicrobial drug, biocide and metal resistance determinants in metagenomic sequence data. *Nucleic Acids Res.* **48**, D561–D569 (2020). 31722416 10.1093/nar/gkz1010 PMC7145535

[32] Rumi, M. A. et al. MetaCompare 2.0: differential ranking of ecological and human health resistome risks. *FEMS Microbiol. Ecol* **100** (2024). 10.1093/femsec/fiae155 PMC12116288 39521944

[33] Katuwal, S., Rafsan, N. A. S., Ashworth, A. J. & Kolar, P. Poultry litter physiochemical characterization based on production conditions for circular systems. *Bioresources* **18**, 3961–3977 (2023).

[34] Ashworth, A. J., Chastain, J. P. & Moore, P. A. Jr. Nutrient characteristics of poultry manure and litter. in Animal Manure 63–87 (American Society of Agronomy, Crop Science Society of America, and Soil Science Society of America, Madison, WI, USA, (2020).

[35] Hoover, N. L., Law, J. Y., Long, L. A. M., Kanwar, R. S. & Soupir, M. L. Long-term impact of poultry manure on crop yield, soil and water quality, and crop revenue. *J. Environ. Manage.* **252**, 109582 (2019). 31614262 10.1016/j.jenvman.2019.109582

[36] Ritz, C. W. & Merka, W. C. Maximizing poultry manure use through nutrient management planning. *UGA Ext. Poult. Scientists*. **1245**, 1–6 (2013).

[37] Męcik, M. et al. Poultry manure-derived microorganisms as a reservoir and source of antibiotic resistance genes transferred to soil autochthonous microorganisms. *J. Environ. Manage.* **348**, 119303 (2023). 37832303 10.1016/j.jenvman.2023.119303

[38] Chinivasagam, H. N., Redding, M., Runge, G. & Blackall, P. J. Presence and incidence of food-borne pathogens in Australian chicken litter. *Br. Poult. Sci.* **51**, 311–318 (2010). 20680865 10.1080/00071668.2010.499424

[39] Wilkinson, K. G., Tee, E., Tomkins, R. B., Hepworth, G. & Premier, R. Effect of heating and aging of poultry litter on the persistence of enteric bacteria. *Poult. Sci.* **90**, 10–18 (2011). 21177438 10.3382/ps.2010-01023

[40] Lanzas, C., Davies, K., Erwin, S. & Dawson, D. On modelling environmentally transmitted pathogens. *Interface Focus*. **10**, 20190056 (2020). 31897293 10.1098/rsfs.2019.0056 PMC6936006

[41] Anttila, J., Ruokolainen, L., Kaitala, V. & Laakso, J. Loss of competition in the outside host environment generates outbreaks of environmental opportunist pathogens. *PLoS One*. **8**, e71621 (2013). 24244752 10.1371/journal.pone.0071621 PMC3752018

[42] Shaji, S., Selvaraj, R. K. & Shanmugasundaram, R. Salmonella infection in poultry: A review on the pathogen and control strategies. *Microorganisms* **11**, 2814 (2023). 38004824 10.3390/microorganisms11112814 PMC10672927

[43] Rohani, P., Breban, R., Stallknecht, D. E. & Drake, J. M. Environmental transmission of low pathogenicity avian influenza viruses and its implications for pathogen invasion. *Proc. Natl. Acad. Sci. U. S. A.* **106**, 10365–10369 (2009). 10.1073/pnas.0809026106 PMC2690603 19497868

[44] Breban, R., Drake, J. M., Stallknecht, D. E. & Rohani, P. The role of environmental transmission in recurrent avian influenza epidemics. *PLoS Comput. Biol.* **5**, e1000346 (2009). 19360126 10.1371/journal.pcbi.1000346 PMC2660440

[45] Oliveira, M., Viñas, I., Usall, J., Anguera, M. & Abadias, M. Presence and survival of Escherichia coli O157:H7 on lettuce leaves and in soil treated with contaminated compost and irrigation water. *Int. J. Food Microbiol.* **156**, 133–140 (2012). 22483400 10.1016/j.ijfoodmicro.2012.03.014

[46] Sanz, C. et al. Implications of the use of organic fertilizers for antibiotic resistance gene distribution in agricultural soils and fresh food products. A plot-scale study. *Sci. Total Environ.* **815**, 151973 (2022). 34843769 10.1016/j.scitotenv.2021.151973

[47] Zhou, S. Y. D. et al. Phyllosphere of staple crops under pig manure fertilization, a reservoir of antibiotic resistance genes. *Environ. Pollut*. **252**, 227–235 (2019). 31153027 10.1016/j.envpol.2019.05.098

[48] U.S. Food and Drug Administration. *Food Safety Modernization Act Final Rule on Produce Safety: Standards for the Growing, Harvesting, Packing, and Holding of Produce for Human Consumption*. (2016).

[49] Pu, C. et al. Exploring the persistence and spreading of antibiotic resistance from manure to biocompost, soils and vegetables. *Sci. Total Environ.* **688**, 262–269 (2019). 31229823 10.1016/j.scitotenv.2019.06.081

[50] Ezugworie, F. N., Okechukwu, V. C. & Onwosi, C. O. Biochar amendment aids in the reduction of antibiotic-resistant bacteria and heavy metals during composting of poultry litter. *Bioremediat. J.* 1–18 (2022).

[51] Lin, M. et al. Challenges of pathogen inactivation in animal manure through anaerobic digestion: a short review. *Bioengineered* **13**, 1149–1161 (2022). 35258411 10.1080/21655979.2021.2017717 PMC8805936

[52] Wang, H. et al. Plant-scale validation of physical heat treatment of poultry litter composts using surrogate and indicator microorganisms for Salmonella. *Appl. Environ. Microbiol.* **87**, AEM.02234-20 (2021). 10.1128/AEM.02234-20 PMC8090882 33355103

[53] Qiu, X., Zhou, G. & Wang, H. Nanoscale zero-valent iron inhibits the horizontal gene transfer of antibiotic resistance genes in chicken manure compost. *J. Hazard. Mater.* **422**, 126883 (2022). 34416685 10.1016/j.jhazmat.2021.126883

[54] Chen, Q. L. et al. Do manure-borne or Indigenous soil microorganisms influence the spread of antibiotic resistance genes in manured soil? *Soil. Biol. Biochem.* **114**, 229–237 (2017).

[55] Forsberg, K. J. et al. Bacterial phylogeny structures soil resistomes across habitats. *Nature* **509**, 612–616 (2014). 24847883 10.1038/nature13377 PMC4079543

[56] Bottery, M. J., Pitchford, J. W. & Friman, V. P. Ecology and evolution of antimicrobial resistance in bacterial communities. *ISME J.* **15**, 939–948 (2021). 33219299 10.1038/s41396-020-00832-7 PMC8115348

[57] University of Wisconsin-Madison. College of Agriculture and Life Sciences. Wisconsin Integrated Cropping Systems Trial. *WICST* https://wicst.wisc.edu (1989–2025).

[58] Posner, J. L., Casler, M. D. & Baldock, J. O. The Wisconsin integrated cropping systems trial: combining agroecology with production agronomy. *Am. J. Altern. Agric.* **10**, 98–107 (1995).

[59] Posner, J. L., Baldock, J. O. & Hedtcke, J. L. Organic and conventional production systems in the Wisconsin integrated cropping systems trials: I. productivity 1990–2002. *Agron. J.* **100**, 253–260 (2008).

[60] Drinkwater, L. E., Friedman, D. & Buck, L. Systems research for agriculture. *Sustainable Agric. Res. Educ. Program* (2016).

[61] Dietz, C. L., Jackson, R. D., Ruark, M. D. & Sanford, G. R. Soil carbon maintained by perennial grasslands over 30 years but lost in field crop systems in a temperate Mollisol. *Commun Earth Environ* **5** (2024).

[62] Rui, Y. et al. Persistent soil carbon enhanced in Mollisols by well-managed grasslands but not annual grain or dairy forage cropping systems. *Proc. Natl. Acad. Sci. U. S. A.* **119**, e2118931119 (2022). 10.1073/pnas.2118931119 PMC8851490 35145033

[63] Sanford, G. R. et al. Soil carbon lost from Mollisols of the North central U.S.A. With 20 years of agricultural best management practices. *Agric. Ecosyst. Environ.* **162**, 68–76 (2012).

[64] Laboski, C. A. M. & Peters, J. B. *Nutrient Application Guidelines for Field, Vegetable, and Fruit Crops in Wisconsin* (2012).

[65] Nutrient and Pest Management Program. Soil Nitrate Tests for Corn Production in Wisconsin: Preplant and Pre-Sideress Nitrate Tests. (2021).

[66] Center for High Throughtput Computing. Center for High Throughput Computing. (2006).

[67] Bolger, A. M., Lohse, M. & Usadel, B. Trimmomatic: a flexible trimmer for illumina sequence data. *Bioinformatics* **30**, 2114–2120 (2014). 24695404 10.1093/bioinformatics/btu170 PMC4103590

[68] Wood, D. E. & Salzberg, S. L. Kraken: ultrafast metagenomic sequence classification using exact alignments. *Genome Biol.* **15**, R46 (2014). 24580807 10.1186/gb-2014-15-3-r46 PMC4053813

[69] Nurk, S., Meleshko, D., Korobeynikov, A. & Pevzner, P. A. MetaSPAdes: a new versatile metagenomic assembler. *Genome Res.* **27**, 824–834 (2017). 28298430 10.1101/gr.213959.116 PMC5411777

[70] Hyatt, D. et al. Prodigal: prokaryotic gene recognition and translation initiation site identification. *BMC Bioinform.* **11**, 119 (2010). 10.1186/1471-2105-11-119 PMC2848648 20211023

[71] Brown, C. L. et al. mobileOG-db: a manually curated database of protein families mediating the life cycle of bacterial mobile genetic elements. *Appl. Environ. Microbiol.* **88**, e0099122 (2022). 36036594 10.1128/aem.00991-22 PMC9499024

[72] R Core Team. R: A Language and Environment for Statistical Computing. (2025).

[73] McMurdie, P. J. & Holmes, S. Phyloseq: an R package for reproducible interactive analysis and graphics of Microbiome census data. *PLoS One*. **8**, e61217 (2013). 23630581 10.1371/journal.pone.0061217 PMC3632530

[74] Paulson, J. N., Stine, O. C., Bravo, H. C. & Pop, M. Differential abundance analysis for microbial marker-gene surveys. *Nat. Methods*. **10**, 1200–1202 (2013). 24076764 10.1038/nmeth.2658 PMC4010126

[75] Oksanen, J., Friendly, M., Blanchet, G. F. & Kindt, R. *Vegan: Community Ecology Package* (2020).

[76] FDA. Summary Report on Antimicrobials Sold or Distributed for Use in Food Producing Animals. (2022).

[77] Lopatkin, A. J. et al. Persistence and reversal of plasmid-mediated antibiotic resistance. *Nat. Commun.* **8**, 1689 (2017). 29162798 10.1038/s41467-017-01532-1 PMC5698434

[78] Bottery, M. J., Wood, A. J. & Brockhurst, M. A. Selective conditions for a multidrug resistanceplasmid depend on the sociality of antibiotic resistance. *Antimicrob. Agents Chemother.* **60**, 2524–2527 (2016). 26787694 10.1128/AAC.02441-15 PMC4808222

[79] Zhang, Y. et al. Exploring the antibiotic resistance genes removal dynamics in chicken manure by composting. *Bioresour Technol.* **410**, 131309 (2024). 39159726 10.1016/j.biortech.2024.131309

[80] Błażejewska, A., Zalewska, M., Grudniak, A. & Popowska, M. A comprehensive study of the microbiome, resistome, and physical and chemical characteristics of chicken waste from intensive farms. *Biomolecules* **12**, 1132 (2022). 36009027 10.3390/biom12081132 PMC9406075

[81] Naumova, N., Barsukov, P., Baturina, O., Rusalimova, O. & Kabilov, M. Addition of chicken litter compost changes bacteriobiome in fallow soil. *Appl. Microbiol. (Basel)*. **4**, 1268–1282 (2024).

[82] Minkina, T. et al. Effect of chicken manure on soil microbial community diversity in poultry keeping areas. *Environ. Geochem. Health*. **45**, 9303–9319 (2023). 36564666 10.1007/s10653-022-01447-x

[83] Rovira, P. et al. Characterization of the microbial resistome in conventional and Raised without antibiotics beef and dairy production systems. *Front. Microbiol.* **10**, 1980 (2019). 31555225 10.3389/fmicb.2019.01980 PMC6736999

