# Supplementary material for: Soil management strategies drive divergent impacts on pathogens and environmental resistomes
Source: Sci Rep. 2025 Dec 5;15:43215. doi: 10.1038/s41598-025-27157-9 (PMC12680746; doi:10.1038/s41598-025-27157-9)
Supplement: Supplementary file 1 — Supplementary Material 1 [file 41598_2025_27157_MOESM1_ESM.docx]

**Supplemental Figures**


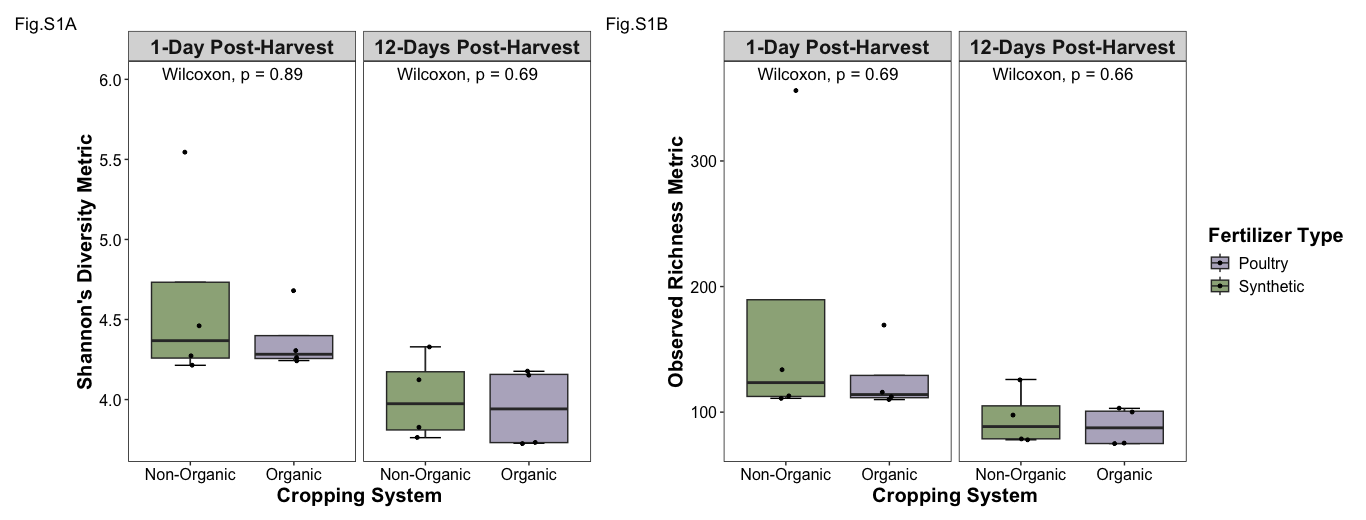


**Fig S1. Resistome alpha diversity within cropping systems**. **S1A)** Antibiotic resistant gene (ARG) Shannon’s diversity was not significantly different 1-day (Wilcoxon test: P = 0.89) or 12-days (Wilcoxon test: P = 0.69) post-harvest between organic and non-organic systems and **S1B)** ARG observed richness was not significantly different 1-day (Wilcoxon test: P = 0.69) or 12-days (Wilcoxon test: P = 0.66) post-harvest between organic and non-organic systems.


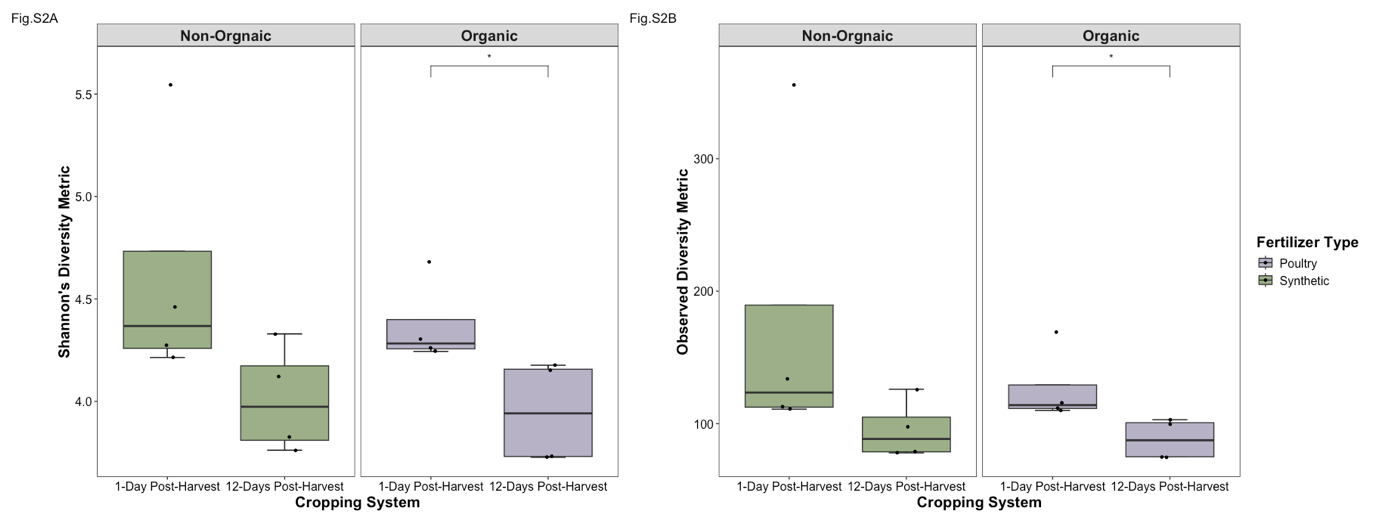


**Fig S2. Resistome alpha diversity within timepoints. S2A)** Antibiotic resistant gene (ARG) Shannon’s diversity was significantly between 1- and 12- days post-harvest within the organic (Wilcoxon test: *P* < 0.05), but not the non-organic system and **S2B)** ARG observed richness was significantly different between 1- and 12- days post-harvest within the organic (Wilcoxon test: *P* < 0.05), but not the non-organic system.


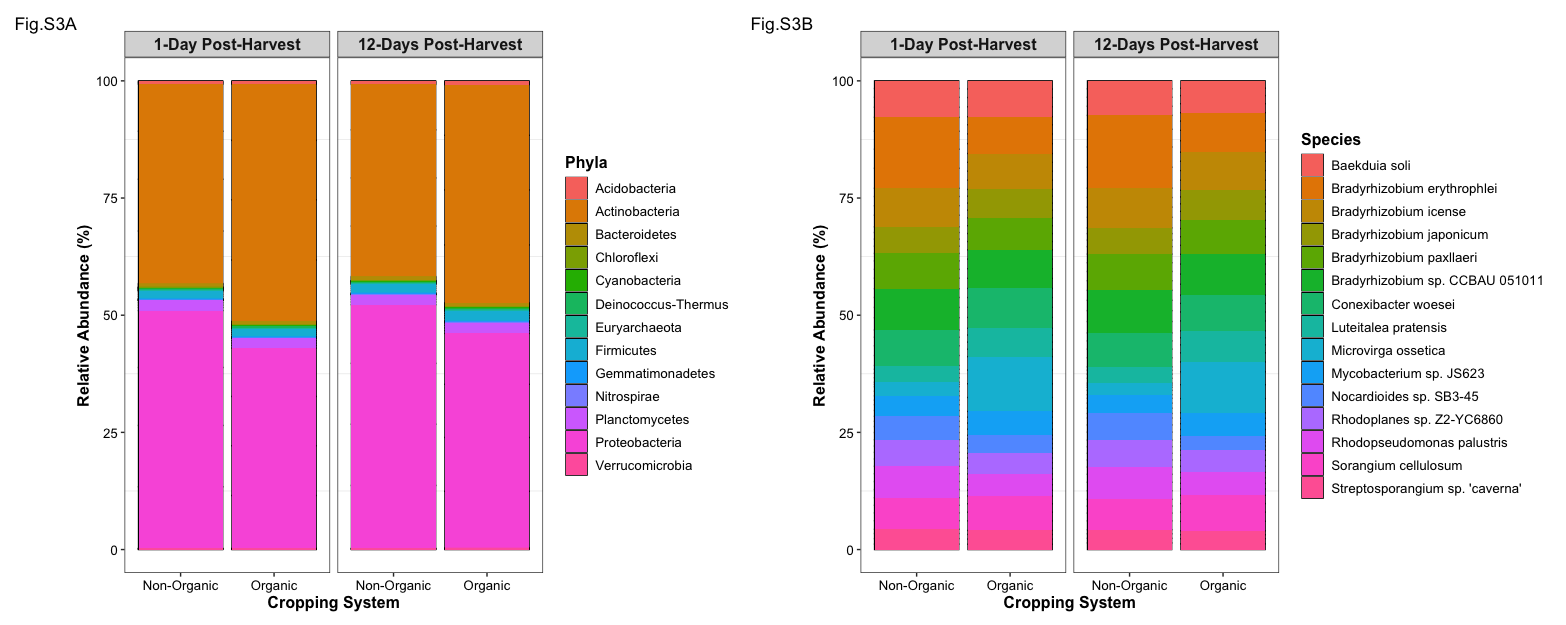


**Fig S3. Relative abundances of top microbial taxa. S3A)** Relative abundance of top 0.1% of phyla showed differences between abundances of phyla, especially Actinobacteria (PERMANOVA: *P* = 0.001) and Proteobacteria (PERMANOVA: *P* = 0.003), between the organic and non-organic systems. **S3B)** Relative abundance of top 0.5% of species showed differences in abundances of the top species between organic and non-organic systems. *Bradyrhizobium erythrophlei,* *Luteitalea pratensis*, *Microvirga ossetica*, *Nocardioides.* sp. SB3-45, *Rhodoplanes* sp. Z2-YC6860, *Rhodopseudomonas palustris*, and Sorangium *cellulosum* abundances were all significantly (PERMANOVA: *P* < 0.005) different between organic and non-organic systems controlled for by time.


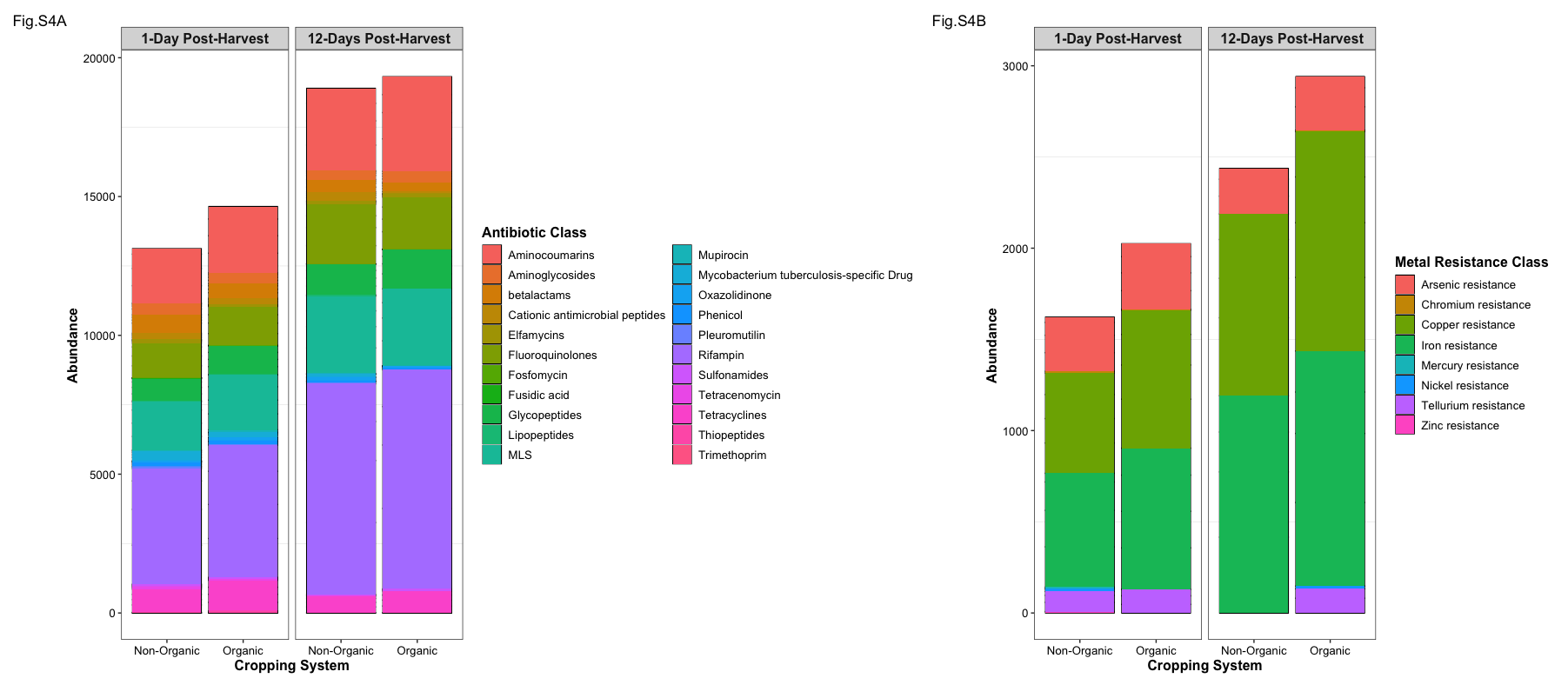


**Fig S4. Normalized abundances of all resistance classes. S4A)** Cumulative sum scaled (CSS) normalized abundance of all antimicrobial resistance classes, showing an increased abundance of total antimicrobial resistance in organic compared to non-organic systems 1- and 12- days post-harvest. **S4B)** CSS normalized abundance of all metal resistance classes, showing an increased abundance of total metal resistance in organic compared to non-organic systems 1- and 12- days post-harvest.


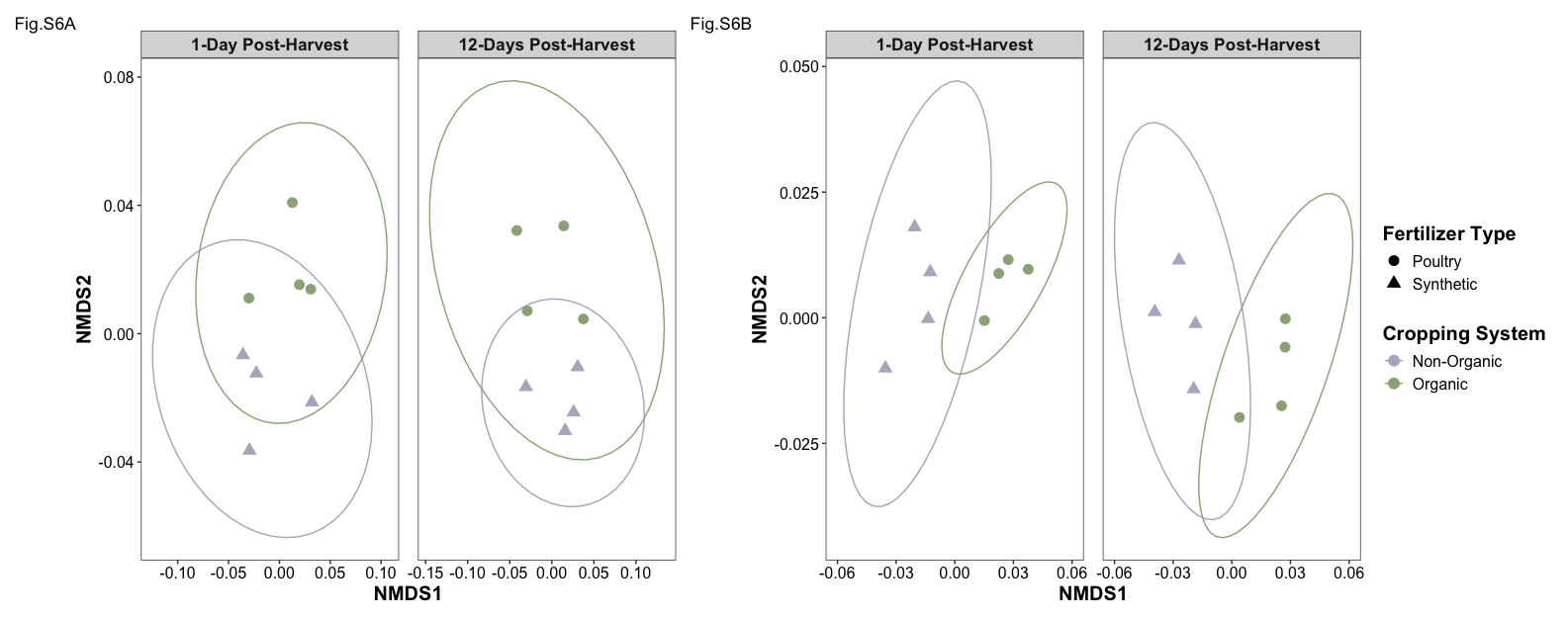


**Fig S6. Beta diversity of the microbiome. S6A)** Non-metric Multidimensional Scaling **(**NMDS) represents similarities in bacterial phyla between non-organic and organic cropping systems 1- and 12- days post-harvest (Stress = 0.173, Non-Metric Fit *R^2^* = 0.977). **S6B)** NMDS represents more dissimilarities in bacterial species between non-organic and organic cropping systems compared to bacterial phyla 1- and 12- days post-harvest (Stress = 0.107, Non-Metric Fit *R^2^* = 0.993).


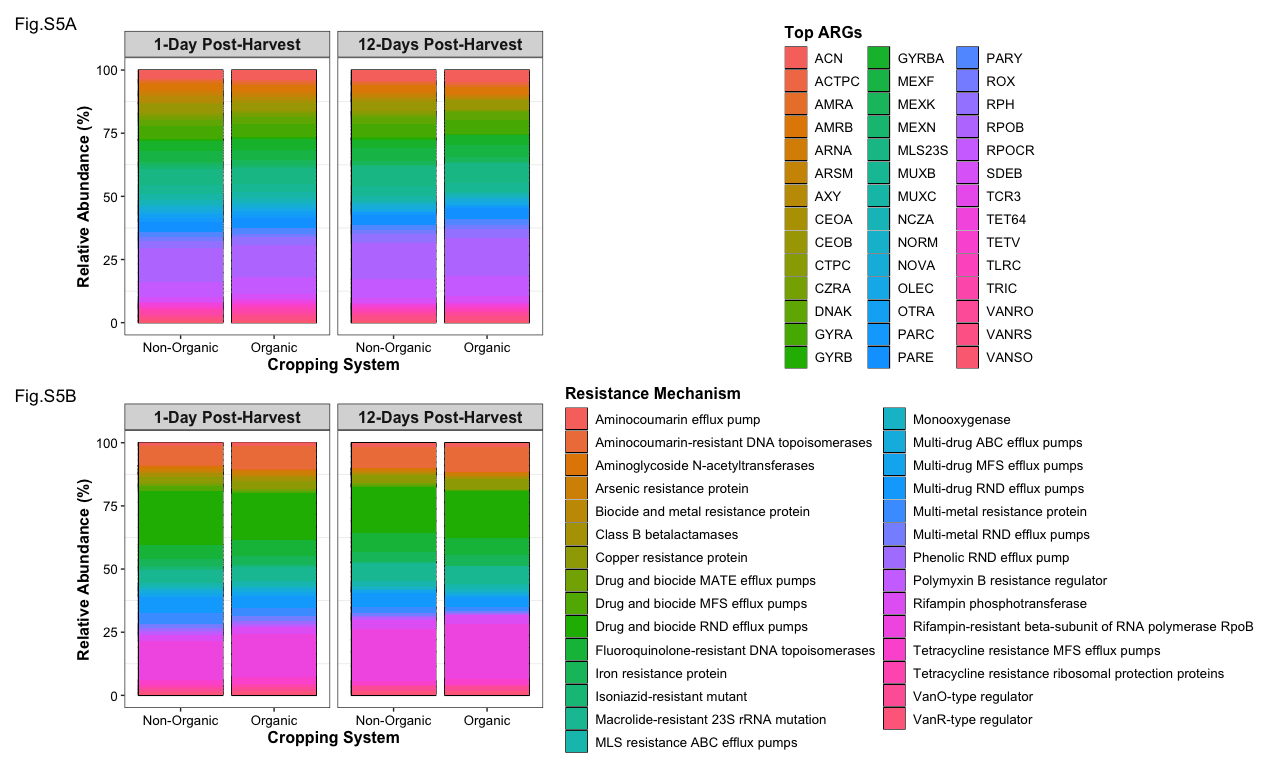


**Fig S5. Relative abundances of top resistance genes and mechanisms. S5A)** Relative abundance of top 0.5% antibiotic resistant genes (ARGs) showed differences in abundances of ARGs between the organic and non-organic systems 1- and 12- days post-harvest. **S5B)** Relative abundance of top 0.5% of resistance mechanisms showed differences in abundances of the top mechanisms between organic and non-organic systems 1- and 12- days post-harvest.


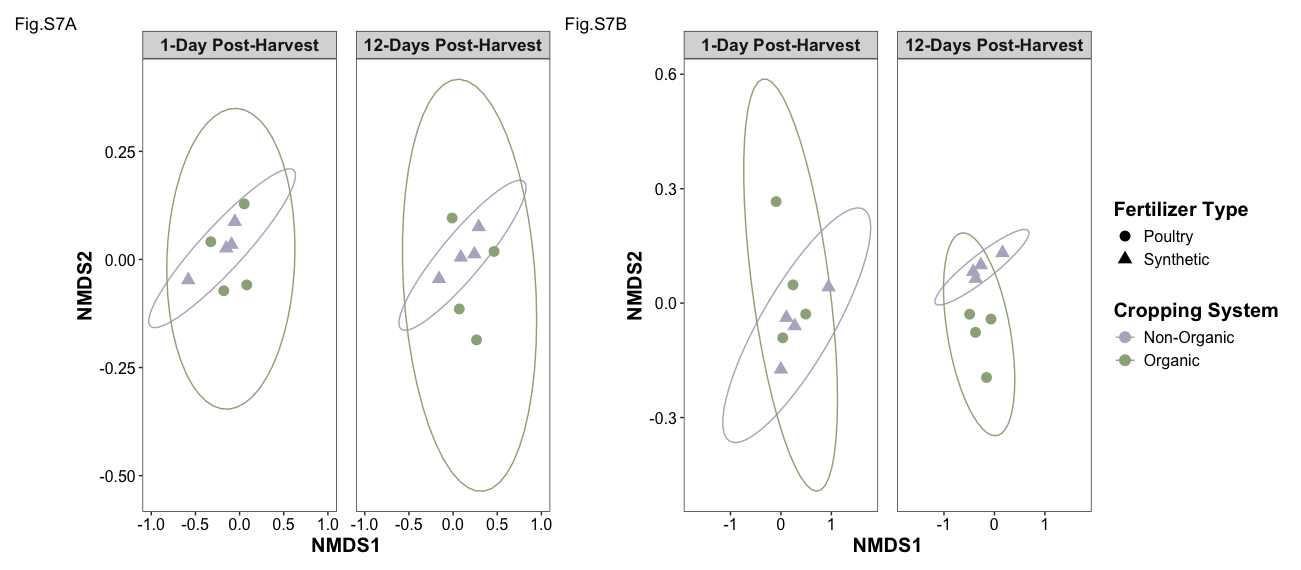


**Fig S7. Beta diversity of the resistome. S7A)** Non-metric multidimensional scaling **(**NMDS) represents similarities in antimicrobial resistance (AMR) classes between non-organic and organic cropping systems 1- and 12- days post-harvest (Stress = 0.061, Non-Metric Fit *R^2^* = 0.996). **S7B)** NMDS represents similarities in AMR mechanisms between non-organic and organic cropping systems; however, timepoint from harvest impacted convergence (Stress = 0.071, Non-Metric Fit *R^2^* *=* 0.995).


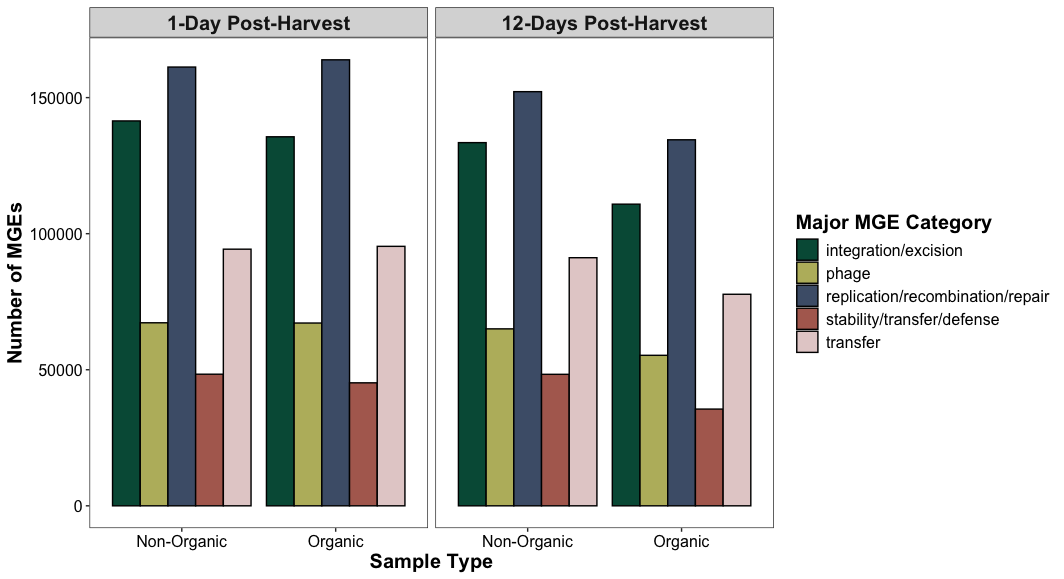


**Fig S8. Mobile genetic element functional categories.** Mobile genetic elements classified by major functional categories for organic and non-organic systems. There was an overall decline MGEs in all categories within the organic system 12-days post-harvest compared to 1-day post-harvest.


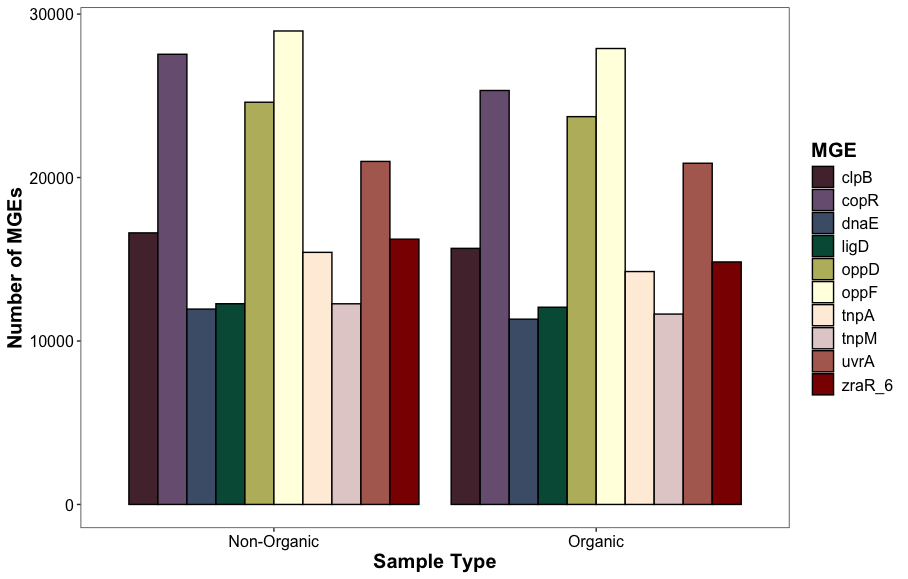


**Fig S9. Top abundant mobile genetic elements.** Top 10 most abundant MGEs identified from organic and non-organic cropping systems. Notably, transposases *tnp*A and *tnp*M, were among this group of top MGEs.


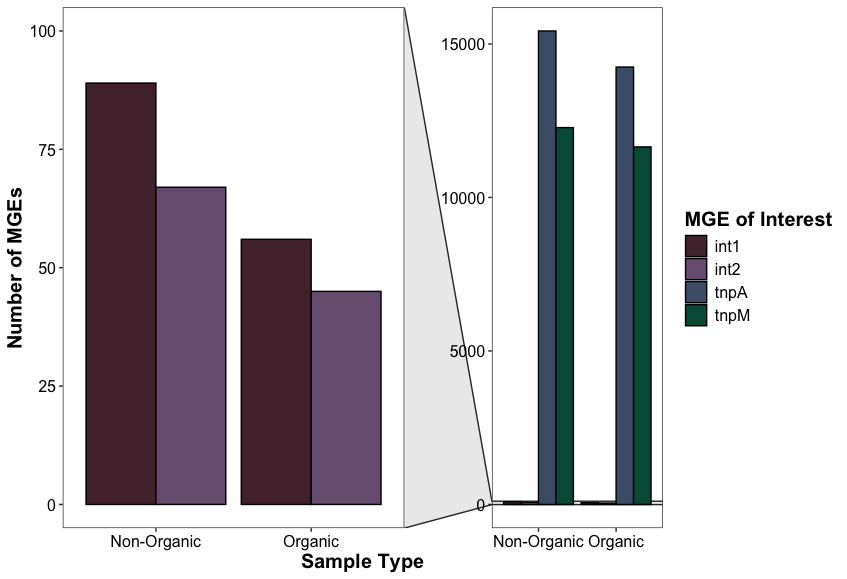


**Fig S10. Mobile Genetic Elements of Interest.** Abundances of integrases, *int*1and *int*2, and transposases, *tnp*A and *tnp*M, of specific interest for horizontal gene transfer. Abundances of transposases were exceedingly higher compared to integrases.


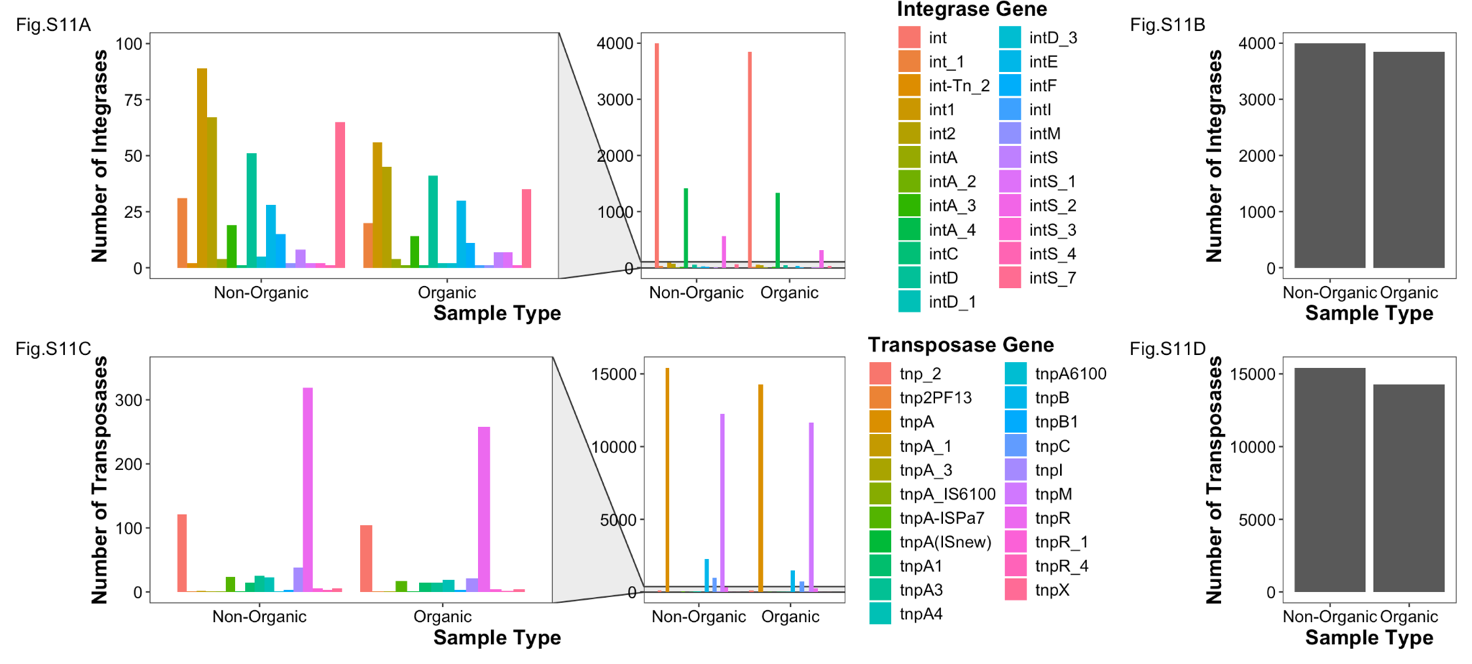


**Fig S11. Integrase and transposase composition. S11A)** Number of individual integrase genes by organic and non-organic cropping system, zoom plots allow visualization of integrases identified at low frequencies. **S11B)** Total number of integrases by organic and non-organic cropping system. **S11C)** Number of individual transposase genes by organic and non-organic cropping system, zoom plots allow visualization of integrases identified at low frequencies. **S11D)** Total number of transposases by organic and non-organic cropping system.
